# Supplementary material for: Bridging the age gap in breast cancer: evaluation of decision support interventions for older women with operable breast cancer: protocol for a cluster randomised controlled trial
Source: BMJ Open. 2017 Jul 31;7(7):e015133. doi: 10.1136/bmjopen-2016-015133 (PMC5642653; doi:10.1136/bmjopen-2016-015133)
Supplement: Author's manuscript [file bmjopen-2016-015133.draft_revisions.pdf]

BMJ Open

Bridging the Age Gap in Breast Cancer: Evaluation of decision support interventions for older women with operable breast cancer: protocol for a cluster randomised controlled trial.

Journal:	*BMJ Open*
Manuscript ID	bmjopen-2016-015133
Article Type:	Protocol
Date Submitted by the Author:	10-Nov-2016
Complete List of Authors:	Collins, Karen; Sheffield Hallam University, Centre for Health and Social Care Research<br>Lifford, Kate; Cochrane Institute of Primary Care & Public Health,<br>Burton, Maria; Sheffield Hallam University, Centre for Health and Social Care Research<br>Reed, Malcolm; Brighton and Sussex Medical School,<br>Edwards, Adrian; Cardiff University, Division of Population Medicine<br>Ring, Alistair; Royal Marsden NHS Foundation Trust<br>Brain, Kate; Cardiff University, Division of Population Medicine<br>Harder, Helena; University of Sussex, SHORE-C Brighton and Sussex Medical School<br>Robinson, Thomas; University of Leicester, Department of Cardiovascular Sciences<br>Cheung, Kwok; University of Nottingham, School of Medicine<br>Morgan, Jenna; University of Sheffield Medical School, Department of Oncology and Metabolism<br>Audisio, Riccardo; University of Liverpool,<br>Ward, Susan; The University of Sheffield, School of Health and Related Research<br>Paul, Richards; The University of Sheffield, School of Health and Related Research<br>Martin, Charleen; University of Sheffield Medical School, Department of Oncology and Metabolism<br>Chater, Tim; The University of Sheffield, School of Health and Related Research<br>Pemberton, Kirsty; The University of Sheffield, School of Health and Related Research<br>Nettleship, Anthony; The University of Sheffield, School of Health and Related Research<br>Murray, Christopher; The University of Sheffield, School of Health and Related Research<br>Walters, Stephen; University of Sheffield, ScHARR<br>Bortolami, Oscar; The University of Sheffield, School of Health and Related Research<br>Armitage, Fiona; University of Sheffield Medical School, Department of Oncology and Metabolism<br>Robert, Leonard; Imperial College Faculty of Medicine<br>Revell, Deirdre; The Unviersity of Sheffield, North Trent Cancer Network Consumer Research Panel

	Gath, Jacqui; Independent Cancer Patients' Voice,<br>Green, Tracy; The Unviersity of Sheffield, North Trent Cancer Network Consumer Research Panel<br>Wyld, Lynda; University of Sheffield Medical School, Department of Oncology and Metabolism
**Primary Subject Heading**:	Oncology
Secondary Subject Heading:	Health services research, Geriatric medicine
Keywords:	CHEMOTHERAPY, Cancer genetics < GENETICS, GERIATRIC MEDICINE, Risk management < HEALTH SERVICES ADMINISTRATION & MANAGEMENT

SCHOLARONE™
Manuscripts

For peer review only

TITLE: Bridging the Age Gap in Breast Cancer: Evaluation of decision support interventions for older women with operable breast cancer: protocol for a cluster randomised controlled trial.

Corresponding author:
Lynda Wyld,
Department of Oncology & Metabolism
The Medical School
Beech Hill Road
Sheffield
S10 2RX
UK
Email: l.wyld@sheffield.ac.uk
Tel: +44 (0) 114 2259066

Karen Collins, Centre for Health and Social Care Research, Sheffield Hallam University, Collegiate Crescent, Sheffield, UK.
Kate Lifford, Division of Population Medicine, School of Medicine, Cardiff University, Neuadd Meirionnydd, Heath Park, Cardiff CF14 4YS, UK
Maria Burton, Centre for Health and Social Care Research, Sheffield Hallam University, Collegiate Crescent, Sheffield, UK.
Malcolm Reed, Brighton and Sussex Medical School, University of Sussex, Falmer, Brighton, UK.
Adrian Edwards, Division of Population Medicine, School of Medicine, Cardiff University, Neuadd Meirionnydd, Heath Park, Cardiff CF14 4YS, UK
Alistair Ring, Royal Marsden Hospital, London.
Katherine Brain, Division of Population Medicine, School of Medicine, Cardiff University, Neuadd Meirionnydd, Heath Park, Cardiff CF14 4YS, UK
Helena Harder, SHORE-C, University of Sussex, Falmer, Brighton, UK.
Thompson Robinson, Department of Cardiovascular Sciences, Leicester Royal Infirmary, Infirmary Square, Leicester, LE2 7LX, UK.
Kwok Leung Cheung, School of Medicine, University of Nottingham, Royal Derby Hospital Centre, Uttoxeter Road, Derby DE22 3DT, UK.
Jenna Morgan, Department of Oncology and Metabolism, University of Sheffield.

For peer review only

Riccardo Audisio, Department of Surgery, University of Liverpool, St Helens Teaching Hospital, Marshalls Cross Road, St Helens, WA9 3DA, UK.

Susan Ward, Department of Health Economics and Decision Science, School for Health and Related Research, University of Sheffield, Sheffield, UK.

Paul Richards, Department of Health Economics and Decision Science, School for Health and Related Research, University of Sheffield, Sheffield, UK.

Charlene Martin, Department of Oncology and Metabolism, University of Sheffield.

Tim Chater, Clinical Trials Research Unit, School for Health and Related Research, University of Sheffield, Sheffield, UK.

Kirsty Pemberton, Clinical Trials Research Unit, School for Health and Related Research, University of Sheffield, Sheffield, UK.

Anthony Nettleship, Epigenesys, School for Health and Related Research, University of Sheffield, Sheffield, UK.

Christopher Murray, Epigenesys, School for Health and Related Research, University of Sheffield, Sheffield, UK.

Stephen Walters, Clinical Trials Research Unit, School for Health and Related Research, University of Sheffield, Sheffield, UK.

Oscar Bortolami, Clinical Trials Research Unit, School for Health and Related Research, University of Sheffield, Sheffield, UK.

Fiona Armitage, University of Sheffield, UK

Robert Leonard, Imperial College Healthcare NHS Trust, St Mary's Hospital London, UK

Jacqui Gath, Deirdre Revell, Tracy Green, Yorkshire and Humberside (formerly North Trent Cancer Network)Consumer Research Panel.

Lynda Wyld, Department of Oncology & Metabolism, University of Sheffield, Sheffield, UK.

Keywords:Breast cancer, decision aid, elderly, primary endocrine therapy, surgery, chemotherapy

Word count excluding title page, abstract, references, figures and tables:3885/4000

For peer review only

ABSTRACT

Introduction

Each year in the UK 16,000 women over the age of 70 develop breast cancer, of whom approximately 6,500 will ultimately die of the disease. Whilst breast cancer outcomes are improving steadily in younger women due to advances in screening and improved therapies, there has been little change in outcomes among the older age group. It is inevitable that co-morbidities/frailty rates are higher, which may increase the risks of some breast cancer treatments such as surgery and chemotherapy, many older women are healthy and may benefit from their use. Adjusting treatment regimens appropriately for age/co-morbidity/frailty is variable and largely non-evidence based, specifically with regard to rates of surgery for operable oestrogen receptor (ER) positive disease and rates of chemotherapy for high-risk disease.

Methods and analysis

This multi-centre, parallel group, pragmatic cluster randomised controlled trial (2015-18), nested with in a larger ongoing “Age Gap Cohort Study” (2012-18; RP-PG-1209-10071), aims to evaluate the effectiveness of a complex intervention of decision support interventions (DESIs) to assist in the treatment decision-making for early breast cancer in older women. The interventions include two patient decision aids (PtDAs) (primary endocrine therapy versus surgery/AET and chemotherapy versus no chemotherapy) and a clinical treatment outcomes algorithm for clinicians.

The primary outcome will be quality of life measured by EORTC QLQ C30. Secondary outcomes will include decision quality, coping, decision regret and treatment allocations

For peer review only

Randomisation is at breast unit level, stratified by high/ low primary endocrine therapy and chemotherapy rates. Women (n=1500) over 70 years with primary operable breast cancer will be recruited and followed up 6 weeks to 2 years post diagnosis with longer term cancer outcomes (overall survival, disease free survival) derived from cancer registry returns. Control arm: no change to usual practice. Intervention arm: usual practice plus DESIs adopted as standard care by clinicians.

Ethics: London South East NHS Research Ethics Committee 12/LO/1808

IRAS reference 115550

Trial registration detail/number:

European Union Drug Regulating Authorities Clinical Trials (EudraCT) number 2015-004220-61

Sponsor's Protocol Code Number Sheffield Teaching Hospitals STH17086

ISRCTN 32447

Strengths and limitations of this study

1. This study has developed two evidence based decision support interventions (DESIs) for women over 70 years diagnosed with breast cancer who are offered a choice of primary endocrine therapy (PET) or surgery (plus adjuvant endocrine therapy, hereafter termed surgery/AET) or chemotherapy versus no chemotherapy.
2. A model of outcomes for older women treated by either PET or surgery/AET or by chemotherapy versusno chemotherapy. These data have been used to construct a web based clinical outcomes management algorithm which allows patient age, co-morbidities, frailty and cancer characteristics to be considered in predicting survival and cancer outcomes.

For peer review only

3. This study will determine the effectiveness of the implementation of the two DESIs on health care outcomes (quality of life, decision quality, coping, decision regret, treatment allocations and short/medium/longer term oncology outcomes).
4. Limitations of the study will potentially be selection bias from recruitment and poor uptake/utilisation of the DESIs at intervention sites, inability to demonstrate a benefit in terms of cancer survival rates without at least 5-10 years follow up or an overall survival advantage due to the competing causes of death in this age group.

INTRODUCTION

Background and rationale

Breast cancer is the most common cancer in women in the UK, with over 53,000 new cases being diagnosed in the UK each year [1]. Of these, 16,000 women will be over the age of 70, a figure which is rising steadily as the UK population ages [2]. Whilst breast cancer outcomes are improving steadily in younger women due to advances in screening and improved therapies, there has been little change in outcomes in this older age group of women. The UK lags significantly behind other European countries in its outcomes for these women. There is a wide variation in practice in the management of breast cancer in older women [3]. The gold standard of care for early breast cancer is surgical removal of the primary cancer (mastectomy or conservation surgery), and diagnostic or therapeutic axillary nodal surgery followed by stage and immunophenotype appropriate adjuvant therapies (chemotherapy, trastuzumab, anti-oestrogens and radiotherapy) to reduce the risks of disease recurrence. There is consistent evidence that older women are less likely to receive surgery, chemotherapy, radiotherapy and trastuzumab, based on the premise that there is less evidence of efficacy and a greater risk of treatment morbidity [4]. In the case of surgery, up to 40% of

For peer review only

older women do not undergo surgery for their breast cancer, andtheir treatment is mainly with anti-oestrogen tablets alone, known as primary endocrine therapy(PET)[5]. Whilst it is inevitable that in older women, co-morbidities and frailty rates are higher, and which will increase the risks of some breast cancer treatments, such as surgery and chemotherapy, many older women are healthy and will benefit in terms of breast cancer outcomes, from their use. Selection of appropriate age, co-morbidity and frailty adjusted treatment regimens is highly variable, largely non-evidence based, and often fails to adequately consider the needs or wishes of patients. Two key areas of local practice variation are rates of surgery for operable oestrogen receptor (ER) positive disease and rates of chemotherapy for high risk disease. PET rates vary four fold between UK centres [3] and are not accounted for by case mix adjustment. Similarly rates of chemotherapy vary 10-fold [4].

Recent reports have advocated the use of PET only in the very old or frail [6]. Current national guidelines state that patients with operable breast cancer should be treated with surgery, and not PET, “irrespective of age” unless this is precluded by co-morbidities [7]; whilst the International Society of Geriatric Oncology (SIOG) and European Society of Breast Cancer Specialists (EUSOMA) recommend that PET should only be offered to patients with a “short estimated life expectancy (less than 2 to 3 years), who are considered unfit for surgery… or who refuse surgery” [8]. However, as a large number of older women are treated with PET in UK and other countries, it is not clear whether this guidance is being followed consistently. PET is associated with high rates of patient satisfaction and low treatment morbidity but in the medium and long term some women may need a change of therapy once anti-oestrogen resistance develops [9]. Randomised trials and a recent Cochrane review have shown that surgery (plus adjuvant anti-oestrogens herein after termed surgery/AET) and PET have equivalent overall survival rates [10-11], although local control rates are superior in surgically treated patients, with disease progression sometimes

For peer review only

necessitating a change of management in patients treated with PET [13-15]. However, for fitter women with a longer predicted life expectancy, there is evidence that breast cancer specific survival rates are inferior with PET [12]. For very frail women where surgery would be unsafe or poorly tolerated, PET is the clear choice in women with oestrogen sensitive disease.

For women at intermediate or higher risk of surgery there is a complex series of trade-offs to be made for each patient. The decision must balance the risks of surgical morbidity (pain, risks associated with hospitalisation, surgical complications) but with a greater certainty of local disease control, against the minimal morbidity with PET but a risk of later local disease progression and the need for a change of treatment to either surgery or alternate anti-oestrogen therapy[13-15].

Chemotherapy utilisation is also very low in women over 70 (14%) [4] and almost non-existent in women over 80, even in those where high phenotypic risk is present (high grade, node positive, ER negative, her-2 positive) [4]. This reflects the fact that whilst there is ample evidence of benefit for chemotherapy in women under 70, most of the randomised trials have upper age cut offs at age 70 or recruit very poorly in this age group, meaning there is little evidence of whether it is effective or not. In addition, there is evidence of an increased risk of significant complications such as neutropenic sepsis in older women [17]. Rates of chemotherapy vary widely between UK breast units, between 6 and 60% in high risk women [16]. This clearly suggests that guidelines for best practice are required. The primary tool used by oncologists to determine the likely benefit of chemotherapy on a patient level basis is Adjuvant! Online [18], although this has been shown to be inaccurate in older women [19]. The more recently developed PREDICT tool [20] performs better in this age group but has limited functionality for taking co-morbidity and frailty into account.

For peer review only

This cluster randomised trial will evaluate the implementation oftwo ("complex") decision support interventions (DESIs) designed to be used by both clinicians and patients to assist in the decision making about treatment for early breast cancer in older women.

The Age Gap Study

The Bridging the Age Gap study [21] is a NIHR funded programme of research (2012-18RP-PG-1209-10071) examining breast cancer management in older women with the ultimate aim of improving outcomes by providing high quality evidence to support treatment decision making in this age group. Two clinical decisions are being studied: the decision relating to the choice of surgery/AET or PET in frailer women with ER positive breast cancer, and the decision regarding use of adjuvant chemotherapy in fitter women with high risk cancers. The study group has developed two decision support interventions (DESIs) based on a systematic evidence summary, expert reference group consultation, patient interviews [22-24] and questionnaires about informational needs and preferences and extensive user- and field-testing with both healthy older women and older women who had faced these decisions. Each DESI includes a clinical management algorithm and two patient decision aids (PtDAs) in the form of a booklet [26, 27] and a (brief) option grid for the clinical decision in question. The clinical management algorithms derive from detailed cancer registry outcome data linked to treatment related morbidity and patient and cancer characteristics from the UK cancer registry (2002-2010) for two UK regions (Northern and Yorkshire and East Midlands) [25]. These online algorithms allow patient age, co-morbidities, frailty and cancer characteristics to be considered by a clinician in predicting survival and cancer outcomes and to help inform breast cancer management decisions for older women [25].

The trial will evaluate these tools in a cluster randomised trial across 46 UK breast units according to the study schematic (Figure 1).

For peer review only

The aims of this study are to evaluate if, how and to what extent, the use of the DESIs embedded as ‘standard of care’ within intervention-arm sites, improves QoL, decision quality (integrating knowledge, attitudes and decision made), coping, illness representations and reduces decision regret, thus indicating improved informed decision making of older women about treatment options for their breast cancer.

To our knowledge this is the first randomised controlled trial to have been undertaken to explore this issue.

Objectives

The objectives are to:

1. To assess the effectiveness of the implementation of DESIs [26][27] in clinical practice in terms of improving patient QoL, decision quality (integrating knowledge, attitudes and decision made), coping, illness representations and reducing decision regret, thus indicating improved informed decision making.
2. To determine if, how, or to what extent, the clinical outcomes management algorithm impacts on clinical decision making among clinicians (change in PET/surgery rates and chemotherapy rates).
3. To determine whether the DESIs are effective in improving short, medium and long term cancer outcomes in this age group of women, (treatment morbidity and overall and disease specific survival).
4. To assess the utility and uptake of the DESIsfrom the perspective of both clinicians and patients by undertaking a formal process evaluation.

Hypotheses

For peer review only

1. Use of the DESIs will improve the quality of life in older women with operable breast cancer and ultimately improve cancer outcomes.
2. Older women faced with a choice of treatment decisions for their breast cancer will report an improved decision quality and shared decision-making experience and less decision regret using DESIs compared to older women who receive usual clinical decision making support.
3. Use of evidence based DESIs will improve short and longer term outcomes by improving treatment personalisation to a woman's health, fitness and cancer characteristics and by improving the quality of decision making, reduce the heterogeneity of practice across the UK.
4. Women in the intervention sites will express more positive illness representations (e.g. increased personal control, positive emotional consequences, less overall threat) and increased use of engagement coping strategies compared to women from the control sites.

METHOD

Study design and setting

This protocol follows the CONSORT statement guidelines for cluster trials [28].

This study is a multi-centre, parallel group, pragmatic cluster randomised controlled trial (2015-18) [29]. It is nested within a larger ongoing Age Gap Cohort Study (2012-18) [21](Figure 1) which is currently recruiting from 46 breast units within in the UK (observational cohort study of current UK management of older women with early breast cancer).

For peer review only

Figure 1: Age Gap study

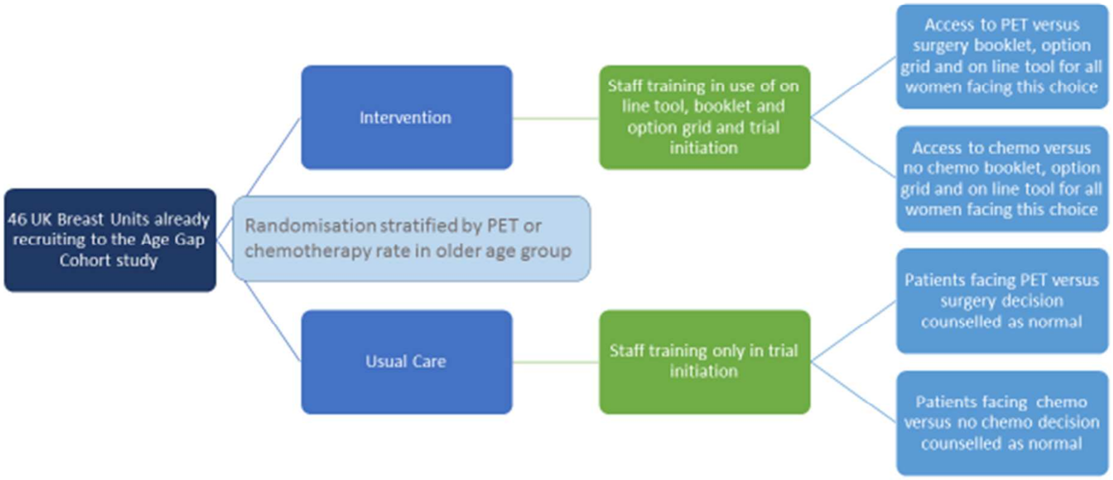

Eligibilitycriteria

Inclusion criteria

(1) Female

(2) Aged over 70 years of age at the time of diagnosis of cancer

(3) Primary operable (TNM categories V7: T1, T2, T3, N0, N1, M0),ER positive invasive breast cancer (core biopsy or diagnostic incision biopsy)

(4) Ability to give informed consent and to read English

Exclusion criteria

(1) Disease unsuitable for surgery e.g. inoperable, locally recurrent or metastatic disease.

(2) Previous invasive breast cancer within the last 5 years.

(3) Non-English speakers

For peer review only

The RCT study

The intervention comprises implementation of a package of two DESIs for the PET versus surgery/AET, or chemotherapy versus no chemotherapy decisions. Each DESIincludes an online algorithm for treatment outcomes, and two patient decision aids (PtDAs)– a booklet and a brief option grid [26-27]. Each DESI is a complex intervention, including training for the clinician in shared decision making and use of the algorithm or PtDAs, and the clinician and patient decide which, if any, of these elements they wish to use to assist the decision making process.

Each online algorithm includes functionality to adjust outcome prediction according to patient age, co-morbidity, frailty, tumour stage and ER status and which gives outputs of 2 and 5 year overall and breast cancer specific survival (Figure 2, illustrating online decision aid outputs).

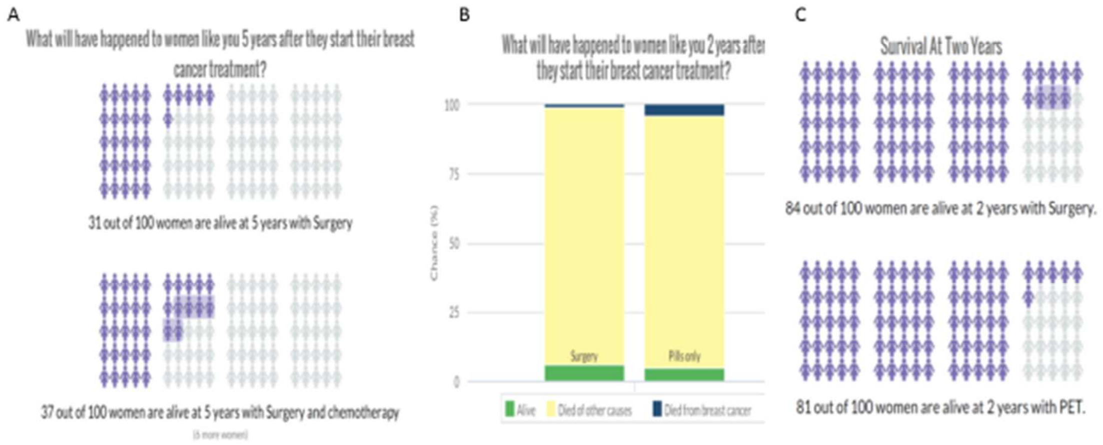

Figure 2. Screen shots from the on line algorithm giving examples of outputs from the tool. A. A fit 70 year old woman with a large node positive ER negative cancer showing the 5 year survival difference of having adjuvant chemotherapy. B, a very frail 93 year old with dementia, cardiac failure and significant dependency showing the relative risks of surgery or PET and C, a moderately fit 82 year old with a strongly ER positive tumour showing the relative risks at 2 years of PET versus surgery.

The algorithms were developed in the earlier phase of the Age Gap Study [25] and weredesigned to guide clinicians and their patients in the treatment of:

(1) frailer older women with ER positive breast cancer to optimise treatment with either PET or surgery/AET,

or

(2) fitter older women who have already had primary surgery and been found to have high risk cancer characteristics (e.g. ER negative, Her 2 positive or node positive breast cancer) to optimise treatment with either adjuvant chemotherapy or no adjuvant chemotherapy (note the term chemotherapy includes chemotherapy +/- trastuzumab if appropriate).

The algorithm is based on a computer model of predicted outcomes and variance caused by patient and disease parameters. Unlike existing web based algorithms for cancer treatment (Adjuvant! OnLine [19] or PREDICT [20]) which do not have the facility to specify frailty or comorbidity in detail (or at all), the Age Gap algorithm permits these factors to be taken into account. The Age Gap tool has been optimised for accuracy in this age group and has been based on analysis of data from over 20 000 UK women over the age of 70 derived from cancer registry data.The algorithm has built in educational materials (including several on line presentations, data sources, FAQs and an animated educational video). The online algorithm is designed to be used by clinicians to guide treatment decision making and also for its outputs to be printed off in a patient facing format that could be used in personalised patient counselling. The report that can be printed off gives specific survival estimates for each treatment option for an individual woman based on her personal and cancer characteristics. This works in much the same way as the print outs from Adjuvant! Online [19] or PREDICT [20] but in this case developed for the PET versus surgery/AET decision and with more detailed data entry relating to the woman's age and fitness level.

For peer review only

Two PtDAs (PET versus surgery/AET [26] and chemotherapy versus no chemotherapy [27]) have been developed during the earlier phase of the study [22-24]. The PtDAs comprise of an option grid [30] and a booklet for each decision (figure 3). The option gridis a one page evidence-based summary of the treatment options alongside patients' frequently asked questions, helping patients to differentiate the key features, risks and benefits of treatment options in relation to their personal values and preferences. The option grid has been designed to be sufficiently brief for use in clinical encounters and accessible enough to support a better dialogue between patients and their clinical team [30]. The booklet provides information about both options including diagrams, side effects and potential risks and benefits. It also includes a section to guide deliberation and encourage the patient to clarify their preferences based around identifying "what is most important to them" [16].

Figure 3. The two patient decision aid booklets for decision support for women facing a PET versus surgery/AET decision [26] or chemotherapy versus no chemotherapy [27] decision.

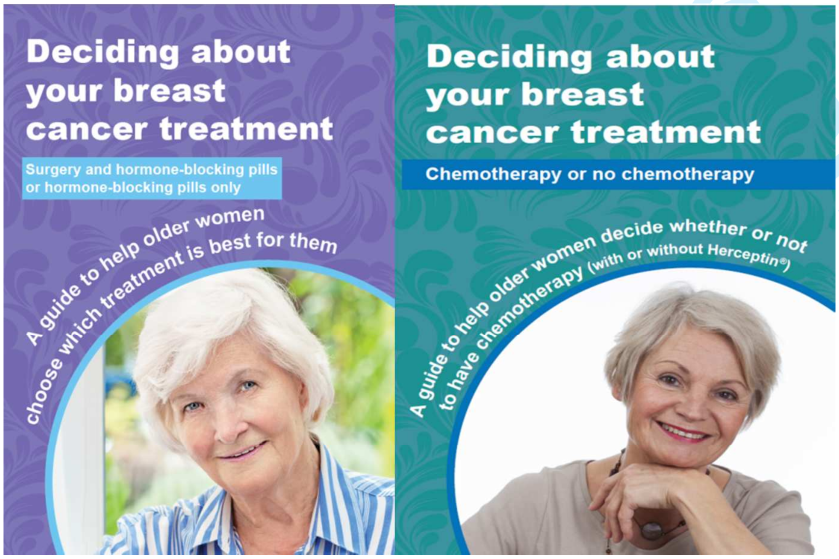

For peer review only

For peer review only - http://bmjopen.bmj.com/site/about/guidelines.xhtml

Data Collection and Outcomes.

Primary outcome measure

The primary outcome measure is global health status/QoL score (questions 29+30 only of The European Organization for Research and Treatment of Cancer QLQ-C30 Reference Manual) (EORTC QLQ-C30) [31] at 6 weeks and 6 months post diagnosis/consent.

Data collection for the study includes detailed information about the patient and their cancer at the time of diagnosis: age, comorbidity (Charlson co-morbidity index [32], frailty- The Barthel Index (ADL) [33] and instrumental activities of daily living scores (IADL) [34]), cognitive status (Mini-mental state examination-MMSE) [35], baseline QoL (EORTC QLQ C30 [31], EORTC breast cancer-specific QoL questionnaire (QLQ-BR23) [36], EORTC QoL questionnaire module for older people with cancer (QLQ-ELD14) [37], EuroQol Group EQ-5D [38]), tumour stage, grade and receptor status. Treatment details are recorded including the type of surgery to the breast and axilla, use of adjuvant therapies (chemotherapy, radiotherapy, trastuzumab and hormonal therapies), including doses and adverse effects recorded using the Common Terminology Criteria for Adverse Events grading system. Follow up is at baseline, 6 weeks, 6, 12, 18 and 24 months after diagnosis/consent. Cancer outcomes, QoL and adverse events are recorded at each visit and in the longer term, women are asked to sign a consent form to permit the trial to collect their Cancer Registry data which will be collected 5 and 10 years following diagnosis and consent to the study. These data will permit us to look at whether using the DESIs alters patterns of treatment decision making between control and intervention sites and whether these impact on long term outcomes. As such this is a uniquely detailed evaluation of such DESIs.

For peer review only

In addition, specific questionnaires relating to patient choice and decision making will be administered. These will apply to all women offered a choice of either PET and surgery/AET or chemotherapy versus no chemotherapy and are administered in relation to the time of their treatment choice.. Secondary outcomes measures here include decision regret (Decision Regret Scale [39], shared decision making (CollaboRATE [40]), patient anxiety (Spielberger short-form State scale of the State-Trait Anxiety Inventory [41], knowledge and preference (knowledge,readiness to decide and preference measure [42-43]), illness perceptions (Brief Illness PerceptionsQuestionnaire [44]) and Coping(brief COPE)[45]).

The timescales for each of these are shown in Table 1.

Table 1.Data items relating to patient-based outcomes and cancer characteristics.

For peer review only

Table 1: Questionnaire Schedule

Standard Age Gap Questionnaires	Baseline	6 weeks	6mths	12mths	18/24 mths	Long-term
IADL	*					
ADL	*					
MMSE	*					
ECOG perf. status	*					
Subjective Global Assessment	*					
Co-morbidity	*					
EQ5D	*	*	*	*	*	
QoL (EORTC-QLQ C30; QLQ-BR23 and (QLQ-ELD14))	*	*	*	*	*	
Decision quality	*	*				
RECIST if PET	*	*	*	*	*	
Registry data access						*
Tissue Access	*					*
Tumour details	*					
Treatment details	*	*	*	*	*	
Adverse events	*	*	*	*	*	
**New for DESI study** (if offered choice of either PETor surgery/AET, or chemotherapy/no chemotherapy)	Baseline (after consent for PET or surgery (AET or after consultation for chemo/no chemo, as applicable)	6 weeks after relevant treatment choice	6 months after relevant treatment choice			
Spielberger Anxiety		*	*			
Collaborate	*					
Decision Regret		*	*			
Knowledgereadiness to decide and preference measures	*					
Brief IPQ		*	*			
Brief COPE		*	*			
**Process evaluation** (if taking part in process evaluation)						
Process evaluation questionnaire		*				

Sample size calculation

The primary endpoint will be the global health status/QoL score (questions 29+30 of EORTC QLQ-C30 [31]) at 6 weeks and 6 months post-diagnosis/consent intervention. Assuming 46units are randomised to either the DESI interventions (25 units) or control (normal care- 25 units) then we can estimate a preliminary sample size assuming a fixed number of clusters (k=46) and attempt to recruit a set number of women per cluster (38). Data from the EORTC Reference Manual [46] suggests a mean Global health status/QoL score of 58.2 with a SD of 25.6 for women aged 70 or more with breast cancer. Cocks and colleagues [47] (suggested the following guidelines for interpreting the Global health status/QoL with estimates for trivial, small, and medium mean differences of 1, 7, and 13 points respectively.

With a standard deviation of 26 points for the Global Health Status/QOL scale we will assume that a mean difference of 7 or more points in Global Health Status/QOL scores between the groups is of clinical/practical importance (a “small” standardised effect size of 0.27). With no allowance for clustering; for the PET versus surgery/AET DESI comparison with 291 eligible women per group we will have 90% power of detecting this difference or more as statistically significant between the groups at the 5% two-sided level. If we assume an intra-class correlation of 0.05 then allowing for the clustered RCT design we will need to recruit 25 women, eligible for the using the decision aids, per cluster (i.e. 50 clusters x 25 women), 1250 in total (this assumes a design effect of 2.2). With a 20% loss to follow-up by six months we need to recruit 34 women per cluster (48 clusters x 32) or 1500 in total (750 per group). Based on our site recruitment data the majority of sites will achievethis number of cases after being open for 24 months.

Randomisation

For peer review only

Randomisation is at breast unit level, stratified by high and low PET and chemotherapy rates. Data for this stratification have been derived from the wider cohort study which has collected data on treatment rates for both PET versus surgery/AET and chemotherapy versus no chemotherapy.

Control arm. Usual standard practice for older women (>70 years) diagnosed with breast cancer with no change to normal treatment decision making practice.

Intervention arm. Usual standard practice for older women (>70 years) diagnosed with breast cancer plus optional clinician and patient access to the package of DESIs which will have been made available to these units to adopt as their standard of care.

In the run in to the trial period (June–Dec 2015), clinical teams (clinicians, research and breast nurses) from the participating sites attended a training event to enhance concordance with the study protocol (control group) and provide additional training on shared decision making and the use of the DESIs (intervention group). This comprised of a 2 hour practical workshopwhich consisted of presentations, demonstrations and discussion based on the MAGIC programme [50].

Recruitment

Potentially eligible women are identified by clinicians and research nursing staff within multi disciplinary teams of the study sites. Study packs are being given to eligible patients either following their clinical consultation where either PET or surgery/AET options or chemotherapy versus no chemotherapy options are discussed.

For peer review only

Data analysis

The statistical analyses will be performed on an intention-to-treat basis comparing the DESI and control groups. All statistical exploratory tests will be two-tailed with p= 0.05. Baseline demographic (e.g. age), physical measurements, and health-related QoL data will be assessed for comparability between the treatment groups. A marginal Generalised Linear Model (GLM), with coefficients estimated using generalised estimating equations (GEE) with robust standard errors and an exchangeable auto correlation matrix in STATA v13 [48-49] will be used to analyse the outcomes and allow for the clustered nature of the data. The exchangeable correlation structure corresponds to an equal correlation model, meaning that the correlations of the outcomes with a cluster, i.e. breast centres, are the same.

For peer review only

For continuous outcomes, such as mean global health status/QoL score (questions 29+30 of EORTC QLQ-C30 [31]) at 6 months post-diagnosis/consent intervention, knowledge score and preference for treatment score, an identity link with a Normal distribution for the outcome will be used. Estimates for the treatment group coefficient from this regression model will be reported along with their associated 95% confidence interval. In the event of differences between the intervention and control groups with respect to baseline demographic, physical, and health-related QoL measurements, then these covariates will be used in the GLM to adjust the treatment effect for these variables. The adjusted regression coefficient estimate for the treatment group parameter along with its 95% confidence interval (CI) will then be reported.

For the other secondary outcomes, at 6 weeks and 6 months, such as the other dimensions of the EORTC QLQ C30 [31], the EORTC QLQ-BR23 [36] and EORTC QLQ-ELD14 [37] the mean QoL dimension scores will be compared between the intervention and control groups, using similar models.

A series of exploratory sub group analyses using a marginal GLM with coefficients estimated using GEE with robust standard errors and an exchangeable auto correlation matrix, with the primary outcome the mean Global health status/QoL score (questions 29+30 of EORTC QLQ-C30 [31]) at 6-month post-diagnosis/consent randomisation as the response will be carried out. An interaction statistical test between the randomised intervention group and subgroup to directly examine the strength of evidence for the treatment difference between the treatment groups (Intervention versus Control) varying between subgroups will be undertaken. Age subgroup (75-79, 80-84, 85-89 and 90+ years) and co-morbidity levels (based on the modified Charlson co-morbidity score [32]) will be the only a priori defined sub groups to be considered for interaction test. Sub group analysis will be performed

For peer review only

regardless of the statistical significance on the overall intervention effect (intervention versus control).

Missing primary outcome data

A sensitivity analysis using a variety of imputation methods, to impute any missing primary outcome data (6-month EORTC QLQ-C30 [31] global health status/QoL score) will be performed. The imputation methods will include last observation carried forward, regression and multiple imputation. The estimates of the treatment effect and its associated confidence interval, from the various imputation methods, will be graphically displayed alongside the results for the observed data.

Process Evaluation

Running alongside the main study, a detailed mixed methods process evaluation is being undertaken at 16 sites to assess the implementation of the DESIs (fidelity to the trial protocol) to consider the DESIs' usefulness and acceptability and examine the facilitators and barriers to embedding them into everyday clinical practice. Arandom selection of breast units was made stratified by trial arm and recruitment rate to the cohort study (high/low PET/surgery/chemo rates).

In summary, the Age Gap study [21] aims to improving outcomes of older women diagnosed with breast cancer by providing high quality evidence to support treatment decision making in this age group. The two evidence based DESIs each include a clinical management algorithm and two patient decision aids (PtDAs) in the form of a booklet and a (brief) option grid for the clinical decision in question. These online algorithmswill allow patient age, co-morbidities, frailty and cancer characteristics to be considered by a clinician in predicting

For peer review only

survival and cancer outcomes and to help inform breast cancer management decisions for older women.

ETHICS AND DISSEMINATION

The study has been fully ethically approved and R and D approval gained at all UK sites.

Acknowledgments

The authors are grateful to all participating sites, and specifically site principal investigators (PI) who have or who are currently contributing to the RCT.

Competing interests: None declared

Funding: This work is supported by National Institute of Health Research Programme Grant [grant number RP-PG-1209-10071]

Contributors LW, KC and MR were overall project leads. Decision aid development and testing KL, MB, KC, LW, MR, AE, KB, JG, DR, FA, HH. Trial management: LW, CM, TC, KP. On line tool development: LW, SW, PR, AN, CM, MB, JM. Statistical advice: SW, OB. Trial management group: LW, MR, KC, TR, KLC, AR, etc. Chemotherapy advisors: AR, RL, HH. All authors have contributed to reading and approved the final manuscript.

Provenance and peer review: Not commissioned; externally peer reviewed

Open access: This is an Open Access article distributed in accordance with the Creative Commons Attribution Non Commercial (CC BY-NC 4.0) license, which permits others to distribute, remix, adapt, build upon this work non-commercially, and license their derivative works on different terms, provided the original work is properly cited and the use is non-commercial. See: http://creativecommons.org/licenses/by-nc/4.0/

For peer review only

REFERENCES

1. Cancer Research UK breast cancer incidence [accessed 1/11/2016] Available from http://www.cancerresearchuk.org/health-professional/cancer-statistics/statistics-by-cancer-type/breast-cancer
2. Office for National Statistics. New cases of cancer diagnosed in England, 2014: selected sites by age group and sex. Office for National Statistics 2010[accessed 1/11/2016]Availablefromhttps://www.ons.gov.uk/peoplepopulationandcommunity/birthsdeathsandmarriages/deaths/bulletins/deathsregistrationsummarytables/previousReleases
3. Morgan J, Richards P, Ward S et al (2015) Case mix analysis and variation in rates of non-surgical treatment of older women with operable breast cancer. *British Journal of Surgery.* 102 (9), 1056-1063.
4. Ring A, Harder H, Langridge C et al. Adjuvant chemotherapy in elderly women with breast cancer (AChEW): an observational study identifying MDT perceptions and barriers to decision making. Ann Oncol. 2013;24(5):1211-9.
5. BCCOM. Breast Cancer Clinical Outcome Measures (BCCOM) Project. Birmingham, UK; 2007
6. Chakrabarti J, Kenny FS, Syed BM, et al. A randomised trial of mastectomy only versus tamoxifen for treating elderly patients with operable primary breast cancer-final results at 20-year follow-up. Crit Rev Oncol Hematol 2011;78:260-264.
7. NICE. Early and locally advanced breast cancer: full guideline.Cardiff: National Collaborating Centre for Cancer; 2009.
8. Biganzoli L, Wildiers H, Oakman C. et al. Management of elderly patients with breast cancer: updated recommendations of the International Society of Geriatric Oncology

(SIOG) and European Society of Breast Cancer Specialists (EUSOMA). Lancet Oncol 2012;13:e148-e160.

9. Morgan JL, Reed MW, Wyld L. Primary endocrine therapy as a treatment for older women with operable breast cancer - a comparison of randomised controlled trial and cohort study findings. Eur J Surg Oncol. 2014;40(6):676-84.

10. Glas de, NA, Jonker JM, Bastiaannet E, et al. Impact of omission of surgery on survival of older patients with breast cancer. Br J Surg. 2014;101(11):1397-404.

11. Hind D, Wyld L, Reed MW. Surgery, with or without tamoxifen, vs tamoxifen alone for older women with operable breast cancer: cochrane review. Br J Cancer. 2007;96(7):1025-9.

12. Morgan J, Wyld L, Collins K, et al. Surgery versus primary endocrine therapy for operable primary breast cancer in elderlywomen (70 years plus). Cochrane Db Syst Rev 2014;5: CD004272.

13. Audisio RA, Wyld L. No standard is set for older women with breast cancer. Eur J Surg Oncol 2015 May; 41 (5): 607

14. Morgan JL, Reed MW, Wyld L. Primary endocrine therapy as a treatment for older women with operable breast cancer - a comparison of randomised controlled trial and cohort study findings. Eur J Surg Oncol. 2014;40(6):676-84.

15. Hille U, Soergel P, Länger F, et al. Aromatase inhibitors as solely treatment in postmenopausal breast cancer patients. Breast J 2012; 18(2):145–50.

16. Elwyn G, Frosch D, Thomson R et al. Shared decision making: a model for clinical practice. J Gen Intern Med 2012 Oct; 27 (10): 1361-7.

17. NICE Clinical guideline. Neutropenic sepsis: prevention and management in people with cancer. CG151]Published date: September 2012

For peer review only

18. Ravdin PM, Siminoff LA, Davis GJ, et al. Computer program to assist in making decisions about adjuvant therapy for women with early breast cancer. J Clin Oncol. 2001;19(4):980-91.

19. deGlas NA, van de Water W, Engelhardt EG, et al. Validity of Adjuvant! Online program in older patients with breast cancer: a population-based study. Lancet Oncol. 2014;15(7):722-9.

20. Wishart GC, Azzato EM, Greenberg DC, et al. PREDICT: a new UK prognostic model that predicts survival following surgery for invasive breast cancer.*Breast Cancer Res. 2010*; 12(1): R1. Published online 2010 January 6. doi: 10.1186/bcr2464. PMCID: PMC2880419

21. Wyld L, Reed MWR, Collins K et al .Bridging the Age Gap in Breast Cancer: Improving outcomes for older women NIHR Programme grant 2012-17RP-PG-1209-10071[Accessed 18/11/ 2016] Available athttp://www.nihr.ac.uk/funding/funded-research/funded-research.htm?postid=2237.

22. Lifford KJ, Witt J, Burton M, et al. Understanding older women's decision making and coping in the context of breast cancer treatment. *BMC Medical Informatics & Decision Making* 2015. 15:45.

23. Burton M,Collins K, Caldon LJM et al.Information Needs of Older Women Faced With a Choice of Primary Endocrine Therapy or Surgery for Early Stage Breast Cancer: A Literature Review; Current Breast Cancer Reports.2014; 6:235–244.

24. Burton M, Collins K, Lifford K et al.The information and decision support needs of older women (>75 yrs) facing treatment choices for breast cancer: a qualitative study (2014). Psycho-Oncology. Published online 22 December 2014.

For peer review only

25. Richards P, Ward S, Morgan J, et al. The use of surgery in the treatment of ER+ early stage breast cancer in England: variation by time, age and patient characteristics. European Journal of Surgical Oncology. 2016; 42(4) 489-96.

26. Deciding about your breast cancer treatment: surgery and hormone-blocking pills or hormone-blocking pills only. Cardiff University, The University of Sheffield, Sheffield Hallam University developed as part of the Bridging the Age Gap Study (RP-PG-1209-10071) August 2015.

27. Deciding about your breast cancer treatment: chemotherapy or no chemotherapy. The University of Sheffield ,Brighton and Sussex Medical School, Sheffield Hallam University and Cardiff Universityas part of the Bridging the Age Gap Study (RP-PG-1209-10071) January 2016.

28. Schulz KF, Altman DG, Moher D, Group C. CONSORT 2010 statement: updated guidelines for reporting parallel group randomized trials. Ann Intern Med. 2010;152(11):726-32.

29. Wyld L, Reed MWR, Collins K on behalf of the Age Gap study. A multi-centre, parallel group, pragmatic cluster randomised controlled trial NIHR Programme grant RP-PG-1209-1007.EUDRACT No:2015-004220-61

30. Marrin k, Brain K, Durand MA, Edwards A et al. . Fast and frugal tools for shared decsion making: How to develop option grids. European Journal for Person Centered Health Care. 2013;1(1):240-5.

31. Aaronson NK, Ahmedzai S, Bergman B, et al. The European Organization for Research and Treatment of Cancer QLQ-C30: a quality-of-life instrument for use in international clinical trials in oncology. J Natl Cancer Inst. 1993;85(5):365-76.

For peer review only

32. Charlson ME, Pompei P, Ales KL, MacKenzie CR. A new method of classifying prognostic comorbidity in longitudinal studies: development and validation. J Chronic Dis. 1987;40(5):373-83.

33. Mahoney FI, Barthel DW. Functional Evaluation: The Barthel Index. Md State Med J. 1965;14:61-5.

34. Lawton MP, Brody EM. Assessment of older people: self-maintaining and instrumental activities of daily living. Gerontologist. 1969;9(3):179-86.

35. Folstein MF, Folstein SE, McHugh PR. "Mini-mental state". A practical method for grading the cognitive state of patients for the clinician. J Psychiatr Res. 1975;12(3):189-98.

36. Sprangers MA, Groenvold M, Arraras JI et al. The European Organization for Research and Treatment of Cancer breast cancer-specific quality-of-life questionnaire module: first results from a three-country field study. J Clin Oncol. 1996;14(10):2756-68.

37. Johnson C, Fitzsimmons D, Gilbert J et al. Development of the European Organisation for Research and Treatment of Cancer quality of life questionnaire module for older people with cancer: The EORTC QLQ-ELD14. Eur J Cancer. 2010;46(12):2242-52.

38. EuroQol Group EuroQol—A new facility for the measurement of health-related quality of life

39. Health Policy, 16 (1990), pp. 199–208.Brehaut JC, O'Connor AM, Wood TJ, Hack TF, Siminoff L, Gordon E, et al. Validation of a decision regret scale. Med Decis Making. 2003;23(4):281-92.

For peer review only

40. Barr PJ, Thompson R, Walsh T, et al. The psychometric properties of CollaboRATE: a fast and frugal patient-reported measure of the shared decision-making process. J Med Internet Res. 2014;16(1):e2.

41. Marteau TM, Bekker H. The development of a six-item short-form of the state scale of the Spielberger State-Trait Anxiety Inventory (STAI). Br J Clin Psychol. 1992;31 (Pt 3):301-6.

42. Sepucha K, Ozanne E, Silvia K, et al. An approach to measuring the quality of breast cancer decisions. Patient Educ Couns. 2007;65(2):261-9.

43. Sepucha KR, Belkora JK, Chang Y, et al. Measuring decision quality: psychometric evaluation of a new instrument for breast cancer surgery. BMC Med Inform Decis Mak. 2012;12:51.

44. Broadbent E, Petrie KJ, Main J, et al. The brief illness perception questionnaire. J Psychosom Res. 2006;60(6):631-7.

45. Carver CS. You want to measure coping but your protocol's too long: Consider the Brief COPE. International Journal of Behavioral Medicine. 1997; 4, 92-100. .

46. Scott NW , Pea F. EORTC QLQ-C30 reference values. EORTC, Brussels. 2008.

47. Cocks K, King MT, Velikova G, et al. Evidence-based guidelines for determination of sample size and interpretation of the European Organisation for the Research and Treatment of Cancer Quality of Life Questionnaire Core 30. J Clin Oncol. 2011;29(1):89-96

48. Campbell MJ & Walters SJ. How to Design, Analyse and Report Cluster Randomised Trials in Medicine and Health Related Research. Wiley and Sons. May 2014

49. StataCorp. 2015. *Stata Statistical Software: Release 14*. College Station, TX: StataCorp LP.

For peer review only

50. Lloyd A & Joseph-Williams N. Case Study: Different outcomes from different approaches – Experience from the Cardiff MAGIC Programme, UK. In Elwyn G, Edwards A, Thompson R (eds.) Shared Decision Making in Health Care. 3rd edition. 2016. Oxford: Oxford University Press.

For peer review only

BMJ Open

Bridging the Age Gap in Breast Cancer: Evaluation of decision support interventions for older women with operable breast cancer: protocol for a cluster randomised controlled trial.

Journal:	*BMJ Open*
Manuscript ID	bmjopen-2016-015133.R1
Article Type:	Protocol
Date Submitted by the Author:	28-Mar-2017

Complete List of Authors:	Collins, Karen; Sheffield Hallam University, Centre for Health and Social Care Research<br>Lifford, Kate; Cochrane Institute of Primary Care & Public Health,<br>Burton, Maria; Sheffield Hallam University, Centre for Health and Social Care Research<br>Reed, Malcolm; Brighton and Sussex Medical School,<br>Edwards, Adrian; Cardiff University, Division of Population Medicine<br>Ring, Alistair; Royal Marsden NHS Foundation Trust<br>Brain, Kate; Cardiff University, Division of Population Medicine<br>Harder, Helena; University of Sussex, SHORE-C Brighton and Sussex Medical School<br>Robinson, Thomas; University of Leicester, Department of Cardiovascular Sciences<br>Cheung, Kwok; University of Nottingham, School of Medicine<br>Morgan, Jenna; University of Sheffield Medical School, Department of Oncology and Metabolism<br>Audisio, Riccardo; University of Liverpool,<br>Ward, Susan; The University of Sheffield, School of Health and Related Research<br>Paul, Richards; The University of Sheffield, School of Health and Related Research<br>Martin, Charleen; University of Sheffield Medical School, Department of Oncology and Metabolism<br>Chater, Tim; The University of Sheffield, School of Health and Related Research<br>Pemberton, Kirsty; The University of Sheffield, School of Health and Related Research<br>Nettleship, Anthony; The University of Sheffield, School of Health and Related Research<br>Murray, Christopher; The University of Sheffield, School of Health and Related Research<br>Walters, Stephen; University of Sheffield, ScHARR<br>Bortolami, Oscar; The University of Sheffield, School of Health and Related Research<br>Armitage, Fiona; University of Sheffield Medical School, Department of Oncology and Metabolism<br>Robert, Leonard; Imperial College Faculty of Medicine<br>Revell, Deirdre; The Unviersity of Sheffield, North Trent Cancer Network Consumer Research Panel

	Gath, Jacqui; Independent Cancer Patients' Voice,<br>Green, Tracy; The Unviersity of Sheffield, North Trent Cancer Network Consumer Research Panel<br>Wyld, Lynda; University of Sheffield Medical School, Department of Oncology and Metabolism
**Primary Subject Heading**:	Oncology
Secondary Subject Heading:	Health services research, Geriatric medicine
Keywords:	CHEMOTHERAPY, Cancer genetics < GENETICS, GERIATRIC MEDICINE, Risk management < HEALTH SERVICES ADMINISTRATION & MANAGEMENT

SCHOLARONE™ Manuscripts

For peer review only

TITLE: Bridging the Age Gap in Breast Cancer: Evaluation of decision support interventions for older women with operable breast cancer: protocol for a cluster randomised controlled trial.

Corresponding author:
Lynda Wyld,
Department of Oncology & Metabolism
The Medical School
Beech Hill Road
Sheffield
S10 2RX
UK

Email: l.wyld@sheffield.ac.uk

Tel: +44 (0) 114 2259066

Karen Collins, Centre for Health and Social Care Research, Sheffield Hallam University, Collegiate Crescent, Sheffield, UK

Malcolm Reed, Brighton and Sussex Medical School, University of Sussex, Falmer, Brighton, UK

Kate Lifford, Division of Population Medicine, School of Medicine, Cardiff University, Neuadd Meirionnydd, Heath Park, Cardiff CF14 4YS, UK

Maria Burton, Centre for Health and Social Care Research, Sheffield Hallam University, Collegiate Crescent, Sheffield, UK.

Malcolm Reed, Brighton and Sussex Medical School, University of Sussex, Falmer, Brighton, UK.

Adrian Edwards, Division of Population Medicine, School of Medicine, Cardiff University, Neuadd Meirionnydd, Heath Park, Cardiff CF14 4YS, UK

Alistair Ring, Royal Marsden Hospital, London.

Katherine Brain, Division of Population Medicine, School of Medicine, Cardiff University, Neuadd Meirionnydd, Heath Park, Cardiff CF14 4YS, UK

Helena Harder, SHORE-C, University of Sussex, Falmer, Brighton, UK.

Thompson Robinson, Department of Cardiovascular Sciences, Leicester Royal Infirmary, Infirmary Square, Leicester, LE2 7LX, UK.

Kwok Leung Cheung, School of Medicine, University of Nottingham, Royal Derby Hospital Centre, Uttoxeter Road, Derby DE22 3DT, UK.

Jenna Morgan, Department of Oncology and Metabolism, University of Sheffield.

For peer review only

Riccardo Audisio, Department of Surgery, University of Liverpool, St Helens Teaching Hospital, Marshalls Cross Road, St Helens, WA9 3DA, UK.

Susan Ward, Department of Health Economics and Decision Science, School for Health and Related Research, University of Sheffield, Sheffield, UK.

Paul Richards, Department of Health Economics and Decision Science, School for Health and Related Research, University of Sheffield, Sheffield, UK.

Charlene Martin, Department of Oncology and Metabolism, University of Sheffield.

Tim Chater, Clinical Trials Research Unit, School for Health and Related Research, University of Sheffield, Sheffield, UK.

Kirsty Pemberton, Clinical Trials Research Unit, School for Health and Related Research, University of Sheffield, Sheffield, UK.

Anthony Nettleship, Epigenesys, School for Health and Related Research, University of Sheffield, Sheffield, UK.

Christopher Murray, Epigenesys, School for Health and Related Research, University of Sheffield, Sheffield, UK.

Stephen Walters, Clinical Trials Research Unit, School for Health and Related Research, University of Sheffield, Sheffield, UK.

Oscar Bortolami, Clinical Trials Research Unit, School for Health and Related Research, University of Sheffield, Sheffield, UK.

Fiona Armitage, University of Sheffield, UK

Robert Leonard, Imperial College Healthcare NHS Trust, St Mary's Hospital London, UK

Jacqui Gath, Deirdre Revell, Tracy Green, Yorkshire and Humberside (formerly North Trent Cancer Network)Consumer Research Panel.

Lynda Wyld, Department of Oncology & Metabolism, University of Sheffield, Sheffield, UK.

Keywords:Breast cancer, decision aid, elderly, primary endocrine therapy, surgery, chemotherapy

Word count excluding title page, abstract, references, figures and tables: 4085/4000

For peer review only

ABSTRACT

Introduction

Each year in the UK 16,000 women over the age of 70 develop breast cancer, of whom approximately 6,500 will ultimately die of the disease. Whilst breast cancer outcomes are improving steadily in younger women due to advances in screening and improved therapies, there has been little change in outcomes among the older age group. It is inevitable that co-morbidities/frailty rates are higher, which may increase the risks of some breast cancer treatments such as surgery and chemotherapy, many older women are healthy and may benefit from their use. Adjusting treatment regimens appropriately for age/co-morbidity/frailty is variable and largely non-evidence based, specifically with regard to rates of surgery for operable oestrogen receptor (ER) positive disease and rates of chemotherapy for high-risk disease.

Methods and analysis

This multi-centre, parallel group, pragmatic cluster randomised controlled trial (2015-18) reported here, is nested within a larger ongoing "Age Gap Cohort Study" (2012-18; RP-PG-1209-10071), aims to evaluate the effectiveness of a complex intervention of decision support interventions (DESIs) to assist in the treatment decision-making for early breast cancer in older women. The interventions include two patient decision aids (PtDAs) (primary endocrine therapy versus surgery/AET and chemotherapy versus no chemotherapy) and a clinical treatment outcomes algorithm for clinicians.

The primary outcome will be quality of life measured by EORTC QLQ C30. Secondary outcomes will include decision quality, coping, decision regret and treatment allocations.

For peer review only

Randomisation is at breast unit level (53 UK sites), stratified by high/ low primary endocrine therapy and chemotherapy rates. Women (n=1500) over 70 years with primary operable breast cancer will be recruited and followed up 6 weeks to 2 years post diagnosis with longer term cancer outcomes (overall survival, disease free survival) derived from cancer registry returns. Control arm: no change to usual practice. Intervention arm: usual practice plus DESIs adopted as standard care by clinicians.

IRAS reference: 115550

Trial registration detail/number:

European Union Drug Regulating Authorities Clinical Trials (EudraCT) number 2015-004220-61

Sponsor's Protocol Code Number Sheffield Teaching Hospitals STH17086

ISRCTN 32447*

*The wider Age Gap study commenced as a cohort study in 2012/13, collecting prospective observational data on older women. At the time there was no requirement for registration on the ISRCTN database as the trial was approved prior to 2013 and was only a cohort study therefore the study team made public notification via the Cancer help database and more recently registered it on the EURDRACT database last year. The trial protocol was changed late 2015/2016 to convert the study to a cluster RCT and at that point registered the revised protocol with the ISRCTN.

For peer review only

Strengths and limitations of this study

- The two evidence based decision support interventions (DESIs) for women over 70 years diagnosed with breast cancer who are offered a choice of primary endocrine therapy (PET) or surgery (plus adjuvant endocrine therapy, hereafter termed surgery/AET) or chemotherapy versus no chemotherapy is, to the best of our knowledge, the first of its kind worldwide.
- The web based clinical outcomes management algorithm is the first of its kind and allows patient age, co-morbidities, frailty and cancer characteristics to be considered in predicting breast cancer survival and cancer outcomes
- A limitations of the trial will potentially be selection bias from recruitment and poor uptake/utilisation of the DESIs at intervention sites
- A second limitation may be an inability to demonstrate a benefit in terms of cancer survival rates without at least 5-10 years follow up or an overall survival advantage due to the competing causes of death in this age group.

For peer review only

INTRODUCTION

Background and rationale

Breast cancer is the most common cancer in women in the UK, with over 53,000 new cases being diagnosed in the UK each year [1].Of these, 16,000 women will be over the age of 70, a figure which is rising steadily as the UK population ages[2]. Whilst breast cancer outcomes are improving steadily in younger women due to advances in screening and improved therapies, there has been little change in outcomes in this older age group of women. The UK lags significantly behind other European countries in its outcomes for these women. There is a wide variation in practice in the management of breast cancer in older women[3]. The gold standard of care for early breast cancer is surgical removal of the primary cancer (mastectomy or conservation surgery), and diagnostic or therapeutic axillary nodal surgery followed by stage and immunophenotype appropriate adjuvant therapies (chemotherapy, trastuzumab, anti-oestrogens and radiotherapy) to reduce the risks of disease recurrence. There is consistent evidence that older women are less likely to receive surgery, chemotherapy, radiotherapy and trastuzumab, based on the premise that there is less evidence of efficacy and a greater risk of treatment morbidity[4]. In the case of surgery, up to 40% of older women do not undergo surgery for their breast cancer, andtheir treatment is mainly with anti-oestrogen tablets alone, known as primary endocrine therapy(PET)[5].Whilst it is inevitable that in older women, co-morbidities and frailty rates are higher, and which will increase the risks of some breast cancer treatments, such as surgery and chemotherapy, many older women are healthy and will benefitin terms of breast cancer outcomes, from their use. Selection of appropriate age, co-morbidity and frailty adjusted treatment regimens is highly variable, largely non-evidence based, and often fails to adequately consider the needs or wishes of patients. Two key areas of local practice variation are rates of surgery for operableoestrogen receptor (ER) positive disease and rates of chemotherapy for high risk

For peer review only

disease. PET rates vary fourfold between UK centres [3] and are not accounted for by case mix adjustment. Similarly rates of chemotherapy vary 10-fold[4].

Recent reports have advocated the use of PET only in the very old or frail[6]. Current national guidelines state that patients with operable breast cancer should be treated with surgery, and not PET, "irrespective of age" unless this is precluded by co-morbidities[7]; whilst the International Society of Geriatric Oncology (SIOG) and European Society of Breast Cancer Specialists (EUSOMA) recommend that PET should only be offered to patients with a "short estimated life expectancy (less than 2 to 3 years), who are considered unfit for surgery… or who refuse surgery"[8]. However, as a large number of older women are treated with PET in UK and other countries, it is not clear whether this guidance is being followed consistently. PET is associated with high rates of patient satisfaction and low treatment morbidity but in the medium and long term some women may need a change of therapy once anti-oestrogen resistance develops [9]. Randomised trials and a recent Cochrane review have shown that surgery (plus adjuvant anti-oestrogens herein after termed surgery/AET) and PET have equivalent overall survival rates [10-11], However, for fitter women with a longer predicted life expectancy, there is evidence that breast cancer specific survival rates are inferior with PET [12]. For very frail women where surgery would be unsafe or poorly tolerated, PET is the clear choice in women with oestrogen sensitive disease [12].

For women at intermediate or higher risk of surgery complications there is a complex series of trade-offs to be made for each patient. The decision must balance the risks of surgical morbidity (pain, risks associated with hospitalisation, surgical complications) but with a greater certainty of local disease control, against the minimal morbidity with PET but a risk of later local disease progression and the need for a change of treatment to either surgery or alternate anti-oestrogen therapy[13-15].

For peer review only

Chemotherapy utilisation is also very low in women over 70 (14%)[4] and almost non-existent in women over 80, even in those where high phenotypic risk is present (high grade, node positive, ER negative, her-2 positive)[4]. Rates of chemotherapy can vary widely between UK breast units, between 6 and 60% in high risk women[16]. This reflects the fact that most of the randomised trials have upper age cut offs at age 70 or recruit very poorly in this age group, meaning there is little evidence of whether it is effective or not. In addition, there is evidence of an increased risk of significant complications such as neutropenic sepsis in older women [17]. This clearly suggests that guidelines for best practice are required. The primary tool used by oncologists to determine the likely benefit of chemotherapy on a patient level basis is Adjuvant!Online[18],although this has been shown to be inaccurate in older women[19].The more recently developed PREDICT tool [20] performs better in this age group but has limited functionality for taking co-morbidity and frailty into account.

This cluster randomised trial will evaluate the implementation of two ("complex") decision support interventions (DESIs) designed to be used by both clinicians and patients to assist in the decision making about treatment for early breast cancer in older women.

The Bridging the Age Gap Study

The Bridging the Age Gap study[21] is a NIHR funded programme of research (2012-18RP-PG-1209-10071) examining breast cancer management in older women with the ultimate aim of improving outcomes by providing high quality evidence to support treatment decision making in this age group.

The study protocol reported here focuses exclusively on the cluster randomised trial part of the wider Bridging the Age Gap Study [21]. The study group has developed two patient facing decision support interventions (DESIs) based on a systematic evidence summary,expert reference group consultation, patient interviews [22-24] and questionnaires

For peer review only

about informational needs and preferences and extensive user- and field-testing with both healthy older women and older women who had faced the decision relating to the choice of surgery/AET or PET in frailer women with ER positive breast cancer, and the decision regarding use of adjuvant chemotherapy in fitter women with high risk cancers. Each DESI includes a clinician facing clinical management algorithm and two patient facing decision aids (PtDAs). The clinician facing management algorithms derive from detailed cancer registry outcome data linked to treatment related morbidity and patient and cancer characteristics from the UK cancer registry (2002-2010) for two UK regions (Northern and Yorkshire and East Midlands) which are representative of the UK population as a whole in terms of demography, population structure and deprivation. This is a large diverse area, representing 23% of the UK population [25]. These online algorithms allow patient age, co-morbidities, frailty and cancer characteristics to be considered by a clinician in predicting survival and cancer outcomes and to help inform breast cancer management decisions for older women [25]. The PtDAs are in the form of a booklet and a (brief) option grid for the clinical decision in question [26,27].

The trial will evaluate these tools in a cluster randomised trial across 53 UK breast units according to the study schematic (Figure 1).

The aims of this trial is to evaluate if, how and to what extent, the use of the DESIs embedded as 'standard of care' within intervention-arm sites, improves QoL, decision quality (integrating knowledge, attitudes and decision made), coping, illness representations and reduces decision regret, thus indicating improved informed decision making of older women about treatment options for their breast cancer.

To our knowledge this is the first randomised controlled trial to have been undertaken to explore this issue.

For peer review only - http://bmjopen.bmj.com/site/about/guidelines.xhtml

Objectives

The objectives are to:

1. To assess the effectiveness of the implementation of DESIs [26][27] in clinical practice in terms of improving patient QoL, decision quality (integrating knowledge, attitudes and decision made), coping, illness representations and reducing decision regret, thus indicating improved informed decision making.
2. To determine if, how, or to what extent, the clinical outcomes management algorithm impacts on clinical decision making among clinicians (change in PET/surgery rates and chemotherapy rates).
3. To determine whether the DESIs are effective in improving short, medium and long term cancer outcomes in this age group of women, (treatment morbidity and overall and disease specific survival).
4. To assess the utility and uptake of the DESIs from the perspective of both clinicians and patients by undertaking a formal process evaluation.

Hypotheses

1. Use of the DESIs will improve the quality of life in older women with operable breast cancer and ultimately improve cancer outcomes.

For peer review only

2. Older women faced with a choice of treatment decisions for their breast cancer will report an improved decision quality and shared decision-making experience and less decision regret using DESIs compared to older women who receive usual clinical decision making support.
3. Use of evidence based DESIs will improve short and longer term outcomes by improving treatment personalisation to a woman's health, fitness and cancer characteristics and by improving the quality of decision making, reduce the heterogeneity of practice across the UK.
4. Women in the intervention sites will express more positive illness representations (e.g. increased personal control, positive emotional consequences, less overall threat) and increased use of engagement coping strategies compared to women from the control sites.

METHOD

Study design and setting

This protocol follows the CONSORT statement guidelines for cluster trials [28].

This study is a multi-centre, parallel group, pragmatic cluster randomised controlled trial (2015-18) [29]. It is nested within a larger ongoing Bridging the Age Gap Cohort Study (2012-18) [21] (Figure 1) which is currently recruiting from 53 breast units within in the UK (observational cohort study of current UK management of older women with early breast cancer).

Figure 1: Overview of the cluster randomised controlled trial

For peer review only

The RCT study

The intervention comprises implementation of a package of two DESIs for the PET versus surgery/AET, or chemotherapy versus no chemotherapy decisions. Each DESI includes an online algorithm for treatment outcomes, and two patient decision aids (PtDAs)– a booklet and a brief option grid [26-27]. Each DESI is a complex intervention, including training for the clinician (breast surgeon, medical oncologist, breast care nurses) on the use of the algorithm (surgeons and medical oncologists only) or PtDAs, and the clinician and patient decide which, if any, of these elements they wish to use to assist the decision making process. The intention being for the intervention to be used as part of everyday clinical practice/pathway within the intervention sites.

Each online algorithm includes functionality to adjust outcome prediction according to patient age, co-morbidity, frailty, tumour stage and ER status and which gives outputs of 2 and 5 year overall and breast cancer specific survival. The algorithms were developed in the earlier phase of the Age Gap Study [25] and were designed to guide clinicians and their patients in the treatment of:

(1) frailer older women with ER positive breast cancer to optimise treatment with either PET or surgery/AET,

or

(2) fitter older women who have already had primary surgery and been found to have high risk cancer characteristics (e.g. ER negative, Her 2 positive or node positive breast cancer) to optimise treatment with either adjuvant chemotherapy or no adjuvant chemotherapy (note the term chemotherapy includes chemotherapy +/- trastuzumab if appropriate).

For peer review only

The algorithm is based on a computer model of predicted outcomes and variance caused by patient and disease parameters. Unlike existing web based algorithms for cancer treatment (Adjuvant! OnLine [19] or PREDICT[20]) which do not have the facility to specify frailty or comorbidity in detail (or at all), the Age Gap algorithm permits these factors to be taken into account. The Age Gap tool has been optimised for accuracy in this age group and has been based on analysis of data from over 20 000 UK women over the age of 70 derived from cancer registry data.The algorithm has built in educational materials (including several on line presentations, data sources, FAQs and an animated educational video). The online algorithm is designed to be used by clinicians to guide treatment decision making and its outputs can be printed off in a patient facing format that could be used in personalised patient counselling. The report provides specific survival estimates for each treatment option for an individual woman based on her personal and cancer characteristics. This works in much the same way as the print outs from Adjuvant!Online[19] or PREDICT[20] but in this case developed for the PET versus surgery/AET decision and with more detailed data entry relating to the woman's age and fitness level.

Two PtDAs (PET versus surgery/AET[26] and chemotherapy versus no chemotherapy [27]) have been developed during the earlier phase of the study [22-24]. The PtDAs comprise of an option grid [30] and a booklet for each decision. The option grid is a one page evidence-based summary of the treatment options alongside patients' frequently asked questions, helping patients to differentiate the key features, risks and benefits of treatment options in relation to their personal values and preferences. The option grid has been designed to be sufficiently brief for use in clinical encounters and accessible enough to support a better dialogue between patients and their clinical team [30]. The booklet provides information about both options including diagrams, side effects and potential risks and benefits. It also

For peer review only

includes a section to guide deliberation and encourage the patient to clarify their preferences based around identifying "what is most important to them" [16].

Eligibility criteria

Inclusion criteria

(1) Female

(2) Aged over 70 years of age at the time of diagnosis of cancer

(3) Primary operable (TNM categories V7: T1, T2, T3, N0, N1, M0), ER positive invasive breast cancer (core biopsy or diagnostic incision biopsy)

(4) Ability to give informed consent and to read English

Exclusion criteria

(1) Disease unsuitable for surgery e.g. inoperable, locally recurrent or metastatic disease.

(2) Previous invasive breast cancer within the last 5 years.

(3) Non-English speakers

Data Collection and Outcomes.

Primary outcome measure

The primary outcome measure for the RCT is global health status/QoL score (questions 29+30 only of The European Organization for Research and Treatment of Cancer QLQ-C30 Reference Manual) (EORTC QLQ-C30) [31] at 6 weeks and 6 months post diagnosis/consent.

For peer review only

An independent data monitoring committee (DMC) comprising of 3 experienced academic clinicians oversees the study and monitors trial conduct and safety and potential harm and has access to all study data. The role being to provide recommendations for trial changes (or closure). Data collection is being undertaken by trained clinical staff within each of the participating sites. The study data manager and study monitor also undertake regular site visits to outline the study protocol, ensure protocol adherence and monitor data collection and completeness. Data collection for the study includes detailed information about the patient and their cancer at the time of diagnosis: age, comorbidity (Charlson co-morbidity index [32], frailty- The Barthel Index (ADL) [33] and instrumental activities of daily living scores (IADL) [34]), cognitive status (Mini-mental state examination-MMSE) [35], baseline QoL (EORTC QLQ C30 [31], EORTC breast cancer-specific QoL questionnaire (QLQ-BR23) [36], EORTC QoL questionnaire module for older people with cancer(QLQ-ELD14) [37], EuroQol Group EQ-5D[38]), tumour stage, grade and receptor status. Treatment details are recorded including the type of surgery to the breast and axilla, use of adjuvant therapies (chemotherapy, radiotherapy, trastuzumab and hormonal therapies), including doses and adverse effects recorded using the Common Terminology Criteria for Adverse Events grading system. Follow up is at baseline, 6 weeks, 6, 12, 18 and 24 months after diagnosis/consent. Cancer outcomes, QoL and adverse events are recorded at each visit and in the longer term, women are asked to sign a consent form to permit the trial to collect their Cancer Registry data which will be collected 5 and 10 years following diagnosis and consent to the study. These data will permit us to look at whether using the DESIs alters patterns of treatment decision making between control and intervention sites and whether these impact on long term outcomes. As such this is a uniquely detailed evaluation of such DESIs.

In addition, specific questionnaires relating to patient choice and decision making will be administered. These will apply to all women offered a choice of either PET and surgery/AET

For peer review only

or chemotherapy versus no chemotherapy and are administered in relation to the time of their treatment choice. Secondary outcomes measures here include decision regret (Decision Regret Scale [39], shared decision making (CollaboRATE [40]), patient anxiety (Spielberger short-form State scale of the State-Trait Anxiety Inventory[41], knowledge and preference (knowledge,readiness to decide and preference measure[42-43]), illness perceptions (Brief Illness Perceptions Questionnaire [44]) and Coping(brief COPE)[45]). Original data collected are entered and kept on file within each of the study sites. This data is entered electronically and stored securely onto password protected databases within local databases and the main trial office. All records that contain names or other personal identifiers, such as locator forms and informed consent forms, are stored separately from study records identified by code number. Only the study steering and DMC have access to the full trial dataset Errors, discrepancies or missing data are captured by the computer programme and the study data manager checks and subsequently follows this up with participating sites.

The timescales for each of these are shown in Table 1.

Table 1.Data items relating to patient-based outcomes and cancer characteristics.

For peer review only

Table 1: Questionnaire Schedule

Standard Age Gap Questionnaires	Baseline	6 weeks	6mths	12mths	18/24 mths	Long-term
IADL	*					
ADL	*					
MMSE	*					
ECOG perf. status	*					
Subjective Global Assessment	*					
Co-morbidity	*					
EQ5D	*	*	*	*	*	
QoL (EORTC-QLQ C30; QLQ-BR23 and (QLQ-ELD14)	*	*	*	*	*	
Decision quality	*	*				
RECIST if PET	*	*	*	*	*	
Registry data access						*
Tissue Access	*					*
Tumour details	*					
Treatment details	*	*	*	*	*	
Adverse events	*	*	*	*	*	
**New for DESI study** (if offered choice of either PETor surgery/AET, or chemotherapy/no chemotherapy)	Baseline (after consent for PET or surgery (AET or after consultation for chemo/no chemo, as applicable)	6 weeks after relevant treatment choice	6 months after relevant treatment choice			
Spielberger Anxiety		*	*			
Collaborate	*					
Decision Regret		*	*			
Knowledgereadiness to decide and preference measures	*					
Brief IPQ		*	*			
Brief COPE		*	*			
**Process evaluation** (if taking part in process evaluation)						
Process evaluation questionnaire		*				

Sample size calculation

The primary endpoint will be the global health status/QoL scale (questions 29 and 30 of the EORTC-QLQ-C30) at 6 months post baseline. Assuming a SD of 21 points for the global health status/QoL scale and a mean difference of 7 or more points on the global health status/QoL scale between the groups is of clinical/practical importance (a "small" standardised effect size of 0.33). With no allowance for clustering; for the PET versus surgery DESI comparison with 190 eligible women per group we will have a 90% power of detecting this difference or more as statistically significant between the groups at the 5% two-sided level. If we assume an intra-class correlation of 0.03 then allowing for the clustered RCT design we will need to recruit 10 women, eligible for using the decision aids, per cluster (i.e. 50 clusters x 10 women), 500 in total (this assumes a design effect of 1.3). With a 20% loss to follow-up by 6 months we need to recruit 13 women per cluster (50 clusters x 13 women) or 650 in total (325 per group).

Randomisation

Randomisation is at breast unit level, stratified by high and low PET and chemotherapy rates. It was therefore not possible to blind the investigators or the study sites to the allocation of participants. Data for this stratification have been derived from the wider cohort study which has collected data on treatment rates for both PET versus surgery/AET and chemotherapy versus no chemotherapy.

Control arm. Usual standard practice for older women (>70 years) diagnosed with breast cancer with no change to normal treatment decision making practice.

For peer review only

Intervention arm. Usual standard practice for older women (>70 years) diagnosed with breast cancer plus optional clinician and patient access to the package of DESIs which will have been made available to these units to adopt as their standard of care.

In the run in to the trial period (June–Dec 2015), clinical teams (clinicians, research and breast nurses) from the participating sites attended a training event to enhance concordance with the study protocol (control group) and provide additional training on shared decision making and the use of the DESIs (intervention group). This comprised of a 2 hour practical workshop which consisted of presentations, demonstrations and discussion based on the MAGIC programme [46].

Recruitment

Potentially eligible women are identified by clinicians (breast surgeons, medical oncologists and specialist breast nurses) and research nursing staff within multi disciplinary teams of the study sites. Study packs are being given to eligible patients either following their clinical consultation where either PET or surgery/AET options or chemotherapy versus no chemotherapy options are discussed. Monthly study newsletters are sent to all participating sites to provide feedback to staff in order to maintain interest and recruitment to the study. Any modifications to the original study protocol will be discussed with the DMEC and approvals sought from the funder and the ethics committee. Recruitment for the trial has now commenced and 750 women have been recruited over the 53 participating sites.

Data analysis

The statistical analyses will be performed on an intention-to-treat basis comparing the DESI and control groups. All statistical exploratory tests will be two-tailed with p= 0.05. Baseline

For peer review only

demographic (e.g. age), physical measurements, and health-related QoL data will be assessed for comparability between the treatment groups. A marginal Generalised Linear Model (GLM), with coefficients estimated using generalised estimating equations (GEE) with robust standard errors and an exchangeable auto correlation matrix in STATA v13 will be used to analyse the outcomes and allow for the clustered nature of the data. The exchangeable correlation structure corresponds to an equal correlation model, meaning that the correlations of the outcomes with a cluster, i.e. breast centres, are the same. For continuous outcomes, such as mean global health status/QoL score (questions 29+30 of EORTC QLQ-C30 [31]) at 6 months post-diagnosis/consent intervention, knowledge score and preference for treatment score, an identity link with a Normal distribution for the outcome will be used. Estimates for the treatment group coefficient from this regression model will be reported along with their associated 95% confidence interval. In the event of differences between the intervention and control groups with respect to baseline demographic, physical, and health-related QoL measurements, then these covariates will be used in the GLM to adjust the treatment effect for these variables. The adjusted regression coefficient estimate for the treatment group parameter along with its 95% confidence interval (CI) will then be reported.

For the other secondary outcomes, at 6 weeks and 6 months, such as the other dimensions of the EORTC QLQ C30 [31], the EORTC QLQ-BR23 [36] and EORTC QLQ-ELD14 [37] the mean QoL dimension scores will be compared between the intervention and control groups, using similar models.

A series of exploratory sub group analyses using a marginal GLM with coefficients estimated using GEE with robust standard errors and an exchangeable auto correlation matrix, with the primary outcome the mean Global health status/QoL score (questions 29+30 of EORTC QLQ-C30 [31]) at 6-month post-diagnosis/consent randomisation as the response will be carried out. An interaction statistical test between the randomised intervention group and

For peer review only

subgroup to directly examine the strength of evidence for the treatment difference between the treatment groups (Intervention versus Control) varying between subgroups will be undertaken. Age subgroup (75-79, 80-84, 85-89 and 90+ years) and co-morbidity levels (based on the modified Charlson co-morbidity score [32]) will be the only a priori defined sub groups to be considered for interaction test. Sub group analysis will be performed regardless of the statistical significance on the overall intervention effect (intervention versus control).

Missing primary outcome data

A sensitivity analysis using a variety of imputation methods, to impute any missing primary outcome data (6-month EORTC QLQ-C30 [31] global health status/QoL score) will be performed. The imputation methods will include last observation carried forward, regression and multiple imputation. The estimates of the treatment effect and its associated confidence interval, from the various imputation methods, will be graphically displayed alongside the results for the observed data.

Process Evaluation

Running alongside the main study, a detailed mixed methods process evaluation is being undertaken at 16 sites to assess the implementation of the DESIs(fidelity to the trial protocol) to consider the DESIs' usefulness and acceptability and examine the facilitators and barriers to embedding them into everyday clinical practice. A random selection of breast units was made stratified by trial arm and recruitment rate to the cohort study (high/low PET/surgery/chemo rates).

In summary, the Age Gap study [21] aims to improving outcomes of older women diagnosed with breast cancer by providing high quality evidence to support treatment decision making

For peer review only

in this age group. The two evidence based DESIs each include a clinical management algorithm and two patient decision aids (PtDAs) in the form of a booklet and a (brief) option grid for the clinical decision in question. These online algorithms will allow patient age, co-morbidities, frailty and cancer characteristics to be considered by a clinician in predicting survival and cancer outcomes and to help inform breast cancer management decisions for older women.

ETHICS AND DISSEMINATION

The study received ethics approval from the London South East NHS Research Ethics Committee (12/LO/1808) and research and development approvals gained from all UK sites. A full study report will be made publicly available once approved by the funders. The findings from the trial will be presented at major scientific conferences and published in international peer reviewed scientific journals.

For peer review only

Acknowledgments

The authors are grateful to all participating sites, and specifically site principal investigators (PI) who have or who are currently contributing to the RCT

Competing interests: None declared

Funding: This work is supported by National Institute of Health Research Programme Grant [grant number RP-PG-1209-10071]

Contributors LW, KC and MR were overall project leads. Decision aid development and testing KL, MB, KC, LW, MR, AE, KB, JG, DR, FA, HH. Trial management: LW, CM, TC, KP. On line tool development: LW, SW, PR, AN, CM, MB, JM. Statistical advice: SW, OB. Trial management group: LW, MR, KC, TR, KLC, AR, etc. Chemotherapy advisors: AR, RL, HH. All authors have contributed to reading and approved the final manuscript.

Provenance and peer review: Not commissioned; externally peer reviewed

Open access: This is an Open Access article distributed in accordance with the Creative Commons Attribution Non Commercial (CC BY-NC 4.0) license, which permits others to distribute, remix, adapt, build upon this work non-commercially, and license their derivative works on different terms, provided the original work is properly cited and the use is non-commercial. See: http://creativecommons.org/licenses/by-nc/4.0/

For peer review only

REFERENCES

1. Cancer Research UK breast cancer incidence [accessed 1/11/2016] Available from http://www.cancerresearchuk.org/health-professional/cancer-statistics/statistics-by-cancer-type/breast-cancer
2. Office for National Statistics. New cases of cancer diagnosed in England, 2014: selected sites by age group and sex. Office for National Statistics 2010[accessed 1/11/2016]Availablefromhttps://www.ons.gov.uk/peoplepopulationandcommunity/birthsdeathsandmarriages/deaths/bulletins/deathsregistrationsummarytables/previousReleases
3. Morgan J, Richards P, Ward S et al (2015) Case mix analysis and variation in rates of non-surgical treatment of older women with operable breast cancer. *British Journal of Surgery.* 102 (9), 1056-1063.
4. Ring A, Harder H, Langridge C et al. Adjuvant chemotherapy in elderly women with breast cancer (AChEW): an observational study identifying MDT perceptions and barriers to decision making. Ann Oncol. 2013;24(5):1211-9.
5. BCCOM. Breast Cancer Clinical Outcome Measures (BCCOM) Project. Birmingham, UK; 2007
6. Chakrabarti J, Kenny FS, Syed BM, et al. A randomised trial of mastectomy only versus tamoxifen for treating elderly patients with operable primary breast cancer-final results at 20-year follow-up. Crit Rev OncolHematol 2011;78:260-264.
7. NICE. Early and locally advanced breast cancer: full guideline.Cardiff: National Collaborating Centre for Cancer; 2009.
8. Biganzoli L, Wildiers H, Oakman C. et al. Management of elderly patients with breast cancer: updated recommendations of the International Society of Geriatric Oncology

For peer review only - http://bmjopen.bmj.com/site/about/guidelines.xhtml

(SIOG) and European Society of Breast Cancer Specialists (EUSOMA). Lancet Oncol 2012;13:e148-e160.

9. Morgan JL, Reed MW, Wyld L. Primary endocrine therapy as a treatment for older women with operable breast cancer - a comparison of randomised controlled trial and cohort study findings. Eur J SurgOncol. 2014;40(6):676-84.

10. Glas de, NA, Jonker JM, Bastiaannet E, et al. Impact of omission of surgery on survival of older patients with breast cancer. Br J Surg. 2014;101(11):1397-404.

11. Hind D, Wyld L, Reed MW. Surgery, with or without tamoxifen, vs tamoxifen alone for older women with operable breast cancer: cochrane review. Br J Cancer. 2007;96(7):1025-9.

12. Morgan J, Wyld L, Collins K, et al. Surgery versus primary endocrine therapy for operable primary breast cancer in elderlywomen (70 years plus). Cochrane Db Syst Rev 2014;5:CD004272.

13. Audisio RA, Wyld L. No standard is set for older women with breast cancer. Eur J SurgOncol 2015 May; 41 (5): 607

14. Morgan JL, Reed MW, Wyld L. Primary endocrine therapy as a treatment for older women with operable breast cancer - a comparison of randomised controlled trial and cohort study findings. Eur J SurgOncol. 2014;40(6):676-84.

15. Hille U, Soergel P, Länger F, et al. Aromatase inhibitorsas solely treatment in postmenopausal breast cancer patients. Breast J 2012;18(2):145–50.

16. Elwyn G, Frosch D, Thomson R et al. Shared decision making: a model for clinical practice. J Gen Intern Med 2012 Oct; 27 (10): 1361-7.

17. NICE Clinical guideline. Neutropenic sepsis: prevention and management in people with cancer. CG151]Published date: September 2012

For peer review only

18. Ravdin PM, Siminoff LA, Davis GJ, et al. Computer program to assist in making decisions about adjuvant therapy for women with early breast cancer. J Clin Oncol. 2001;19(4):980-91.

19. deGlas NA, van de Water W, Engelhardt EG, et al. Validity of Adjuvant! Online program in older patients with breast cancer: a population-based study. Lancet Oncol. 2014;15(7):722-9.

20. Wishart GC, Azzato EM, Greenberg DC, et al. PREDICT: a new UK prognostic model that predicts survival following surgery for invasive breast cancer.*Breast Cancer Res. 2010*; 12(1): R1. Published online 2010 January 6. doi: 10.1186/bcr2464. PMCID: PMC2880419

21. Wyld L, Reed MWR, Collins K et al .Bridging the Age Gap in Breast Cancer: Improving outcomes for older women NIHR Programme grant 2012-17RP-PG-1209-10071[Accessed 18/11/ 2016] Available athttp://www.nihr.ac.uk/funding/funded-research/funded-research.htm?postid=2237.

22. Lifford KJ, Witt J, Burton M, et al. Understanding older women's decision making and coping in the context of breast cancer treatment. *BMC Medical Informatics & Decision Making* 2015. 15:45.

23. Burton M,Collins K, Caldon LJM et al.Information Needs of Older Women Faced With a Choice of Primary Endocrine Therapy or Surgery for Early Stage Breast Cancer: A Literature Review; Current Breast Cancer Reports.2014; 6:235–244.

24. Burton M, Collins K, Lifford K et al.The information and decision support needs of older women (>75 yrs) facing treatment choices for breast cancer: a qualitative study (2014). Psycho-Oncology. Published online 22 December 2014.

For peer review only

25. Richards P, Ward S, Morgan J, et al. The use of surgery in the treatment of ER+ early stage breast cancer in England: variation by time, age and patient characteristics. European Journal of Surgical Oncology. 2016; 42(4) 489-96.

26. Deciding about your breast cancer treatment: surgery and hormone-blocking pills or hormone-blocking pills only. Cardiff University, The University of Sheffield, Sheffield Hallam University developed as part of the Bridging the Age Gap Study (RP-PG-1209-10071) August 2015.

27. Deciding about your breast cancer treatment: chemotherapy or no chemotherapy. The University of Sheffield ,Brighton and Sussex Medical School, Sheffield Hallam University and Cardiff Universityas part of the Bridging the Age Gap Study (RP-PG-1209-10071) January 2016.

28. Schulz KF, Altman DG, Moher D, Group C. CONSORT 2010 statement: updated guidelines for reporting parallel group randomized trials. Ann Intern Med. 2010;152(11):726-32.

29. Wyld L, Reed MWR, Collins K on behalf of the Age Gap study. A multi-centre, parallel group, pragmatic cluster randomised controlled trial NIHR Programme grant RP-PG-1209-1007.EUDRACT No:2015-004220-61

30. Marrin k, Brain K, Durand MA, Edwards A et al. . Fast and frugal tools for shared decsion making: How to develop option grids.European Journal for Person Centered Health Care. 2013;1(1):240-5.

31. Aaronson NK, Ahmedzai S, Bergman B, et al. The European Organization for Research and Treatment of Cancer QLQ-C30: a quality-of-life instrument for use in international clinical trials in oncology. J Natl Cancer Inst. 1993;85(5):365-76.

For peer review only

32. Charlson ME, Pompei P, Ales KL, MacKenzie CR. A new method of classifying prognostic comorbidity in longitudinal studies: development and validation. J Chronic Dis. 1987;40(5):373-83.

33. Mahoney FI, Barthel DW. Functional Evaluation: The Barthel Index. Md State Med J. 1965;14:61-5.

34. Lawton MP, Brody EM. Assessment of older people: self-maintaining and instrumental activities of daily living. Gerontologist. 1969;9(3):179-86.

35. Folstein MF, Folstein SE, McHugh PR. "Mini-mental state". A practical method for grading the cognitive state of patients for the clinician. J Psychiatr Res. 1975;12(3):189-98.

36. Sprangers MA, Groenvold M, Arraras JI et al. The European Organization for Research and Treatment of Cancer breast cancer-specific quality-of-life questionnaire module: first results from a three-country field study. J Clin Oncol. 1996;14(10):2756-68.

37. Johnson C, Fitzsimmons D, Gilbert J et al. Development of the European Organisation for Research and Treatment of Cancer quality of life questionnaire module for older people with cancer: The EORTC QLQ-ELD14. Eur J Cancer. 2010;46(12):2242-52.

38. EuroQol Group EuroQol—A new facility for the measurement of health-related quality of life

39. Health Policy, 16 (1990), pp. 199–208.Brehaut JC, O'Connor AM, Wood TJ, Hack TF, Siminoff L, Gordon E, et al. Validation of a decision regret scale. Med Decis Making. 2003;23(4):281-92.

For peer review only

40. Barr PJ, Thompson R, Walsh T, et al. The psychometric properties of CollaboRATE: a fast and frugal patient-reported measure of the shared decision-making process. J Med Internet Res. 2014;16(1):e2.

41. Marteau TM, Bekker H. The development of a six-item short-form of the state scale of the Spielberger State-Trait Anxiety Inventory (STAI). Br J Clin Psychol. 1992;31 (Pt 3):301-6.

42. Sepucha K, Ozanne E, Silvia K, et al. An approach to measuring the quality of breast cancer decisions. Patient Educ Couns. 2007;65(2):261-9.

43. Sepucha KR, Belkora JK, Chang Y, et al. Measuring decision quality: psychometric evaluation of a new instrument for breast cancer surgery. BMC Med Inform Decis Mak. 2012;12:51.

44. Broadbent E, Petrie KJ, Main J, et al. The brief illness perception questionnaire. J Psychosom Res. 2006;60(6):631-7.

45. Carver CS. You want to measure coping but your protocol's too long: Consider the Brief COPE. International Journal of Behavioral Medicine.1997; 4, 92-100. .

46. Lloyd A & Joseph-Williams N. Case Study: Different outcomes from different approaches – Experience from the Cardiff MAGIC Programme, UK. In Elwyn G, Edwards A, Thompson R (eds.) Shared Decision Making in Health Care. 3rd edition. 2016. Oxford: Oxford University Press.

For peer review only

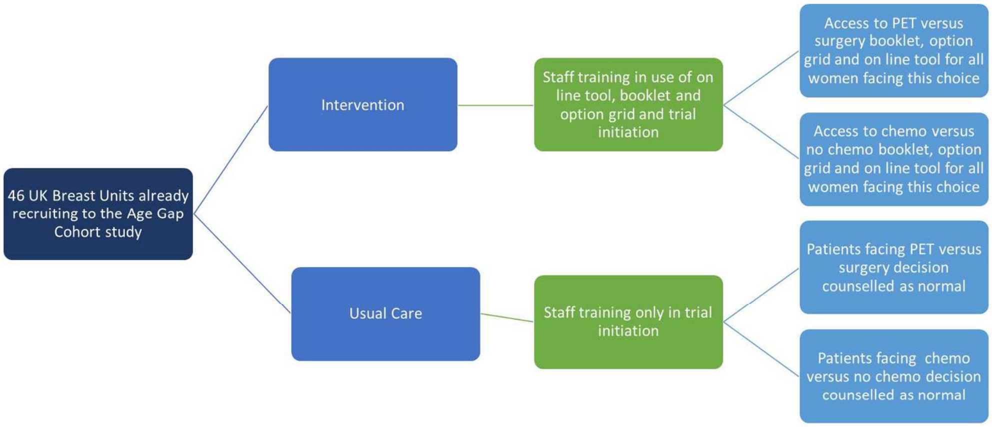

Figure 1: Overview of the Cluster randomised Controlled Trial

335x182mm (300 x 300 DPI)

review only

STANDARD PROTOCOL ITEMS: RECOMMENDATIONS FOR INTERVENTIONAL TRIALS

SPIRIT 2013 Checklist: Recommended items to address in a clinical trial protocol and related documents*

Section/item	Item No	Description	Addressed on page number
**Administrative information**			
Title	1	Descriptive title identifying the study design, population, interventions, and, if applicable, trial acronym	1
Trial registration	2a	Trial identifier and registry name. If not yet registered, name of intended registry	4
	2b	All items from the World Health Organization Trial Registration Data Set	Within main protocol but N/A for journal
Protocol version	3	Date and version identifier	Within main protocol but N/A for journal
Funding	4	Sources and types of financial, material, and other support	23
Roles and responsibilities	5a	Names, affiliations, and roles of protocol contributors	23
	5b	Name and contact information for the trial sponsor	4
	5c	Role of study sponsor and funders, if any, in study design; collection, management, analysis, and interpretation of data; writing of the report; and the decision to submit the report for publication, including whether they will have ultimate authority over any of these activities	4

For peer review only

For peer review only - http://bmjopen.bmj.com/site/about/guidelines.xhtml

	5d	Composition, roles, and responsibilities of the coordinating centre, steering committee, endpoint adjudication committee, data management team, and other individuals or groups overseeing the trial, if applicable (see Item 21a for data monitoring committee)	Within main protocol but N/A for journal
**Introduction**			
Background and rationale	6a	Description of research question and justification for undertaking the trial, including summary of relevant studies (published and unpublished) examining benefits and harms for each intervention	6-8
	6b	Explanation for choice of comparators	18,19
Objectives	7	Specific objectives or hypotheses	10-11
Trial design	8	Description of trial design including type of trial (eg, parallel group, crossover, factorial, single group), allocation ratio, and framework (eg, superiority, equivalence, noninferiority, exploratory)	11
**Methods: Participants, interventions, and outcomes**			
Study setting	9	Description of study settings (eg, community clinic, academic hospital) and list of countries where data will be collected. Reference to where list of study sites can be obtained	11
Eligibility criteria	10	Inclusion and exclusion criteria for participants. If applicable, eligibility criteria for study centres and individuals who will perform the interventions (eg, surgeons, psychotherapists)	14
Interventions	11a	Interventions for each group with sufficient detail to allow replication, including how and when they will be administered	12-13
	11b	Criteria for discontinuing or modifying allocated interventions for a given trial participant (eg, drug dose change in response to harms, participant request, or improving/worsening disease)	15
	11c	Strategies to improve adherence to intervention protocols, and any procedures for monitoring adherence (eg, drug tablet return, laboratory tests)	19
	11d	Relevant concomitant care and interventions that are permitted or prohibited during the trial	N/A

For peer review only - http://bmjopen.bmj.com/site/about/guidelines.xhtml

Outcomes	12	Primary, secondary, and other outcomes, including the specific measurement variable (eg, systolic blood pressure), analysis metric (eg, change from baseline, final value, time to event), method of aggregation (eg, median, proportion), and time point for each outcome. Explanation of the clinical relevance of chosen efficacy and harm outcomes is strongly recommended	15-17
Participant timeline	13	Time schedule of enrolment, interventions (including any run-ins and washouts), assessments, and visits for participants. A schematic diagram is highly recommended (see Figure)	17
Sample size	14	Estimated number of participants needed to achieve study objectives and how it was determined, including clinical and statistical assumptions supporting any sample size calculations	18
Recruitment	15	Strategies for achieving adequate participant enrolment to reach target sample size	19
**Methods: Assignment of interventions (for controlled trials)**			
Allocation:			
Sequence generation	16a	Method of generating the allocation sequence (eg, computer-generated random numbers), and list of any factors for stratification. To reduce predictability of a random sequence, details of any planned restriction (eg, blocking) should be provided in a separate document that is unavailable to those who enrol participants or assign interventions	18
Allocation concealment mechanism	16b	Mechanism of implementing the allocation sequence (eg, central telephone; sequentially numbered, opaque, sealed envelopes), describing any steps to conceal the sequence until interventions are assigned	N/A 18
Implementation	16c	Who will generate the allocation sequence, who will enrol participants, and who will assign participants to interventions	18
Blinding (masking)	17a	Who will be blinded after assignment to interventions (eg, trial participants, care providers, outcome assessors, data analysts), and how	N/A 18
	17b	If blinded, circumstances under which unblinding is permissible, and procedure for revealing a participant’s allocated intervention during the trial	N/A
**Methods: Data collection, management, and analysis**			

For peer review only - http://bmjopen.bmj.com/site/about/guidelines.xhtml

Data collection methods	18a	Plans for assessment and collection of outcome, baseline, and other trial data, including any related processes to promote data quality (eg, duplicate measurements, training of assessors) and a description of study instruments (eg, questionnaires, laboratory tests) along with their reliability and validity, if known. Reference to where data collection forms can be found, if not in the protocol	14-16
	18b	Plans to promote participant retention and complete follow-up, including list of any outcome data to be collected for participants who discontinue or deviate from intervention protocols	17
Data management	19	Plans for data entry, coding, security, and storage, including any related processes to promote data quality (eg, double data entry; range checks for data values). Reference to where details of data management procedures can be found, if not in the protocol	16
Statistical methods	20a	Statistical methods for analysing primary and secondary outcomes. Reference to where other details of the statistical analysis plan can be found, if not in the protocol	19-21
	20b	Methods for any additional analyses (eg, subgroup and adjusted analyses)	21
	20c	Definition of analysis population relating to protocol non-adherence (eg, as randomised analysis), and any statistical methods to handle missing data (eg, multiple imputation)	21
**Methods: Monitoring**			
Data monitoring	21a	Composition of data monitoring committee (DMC); summary of its role and reporting structure; statement of whether it is independent from the sponsor and competing interests; and reference to where further details about its charter can be found, if not in the protocol. Alternatively, an explanation of why a DMC is not needed	15
	21b	Description of any interim analyses and stopping guidelines, including who will have access to these interim results and make the final decision to terminate the trial	17
Harms	22	Plans for collecting, assessing, reporting, and managing solicited and spontaneously reported adverse events and other unintended effects of trial interventions or trial conduct	15
Auditing	23	Frequency and procedures for auditing trial conduct, if any, and whether the process will be independent from investigators and the sponsor	15,17
**Ethics and dissemination**			

For peer review only - http://bmjopen.bmj.com/site/about/guidelines.xhtml

Research ethics approval	24	Plans for seeking research ethics committee/institutional review board (REC/IRB) approval	22
Protocol amendments	25	Plans for communicating important protocol modifications (eg, changes to eligibility criteria, outcomes, analyses) to relevant parties (eg, investigators, REC/IRBs, trial participants, trial registries, journals, regulators)	15
Consent or assent	26a	Who will obtain informed consent or assent from potential trial participants or authorised surrogates, and how (see Item 32)	19
	26b	Additional consent provisions for collection and use of participant data and biological specimens in ancillary studies, if applicable	N/A
Confidentiality	27	How personal information about potential and enrolled participants will be collected, shared, and maintained in order to protect confidentiality before, during, and after the trial	16
Declaration of interests	28	Financial and other competing interests for principal investigators for the overall trial and each study site	23
Access to data	29	Statement of who will have access to the final trial dataset, and disclosure of contractual agreements that limit such access for investigators	15
Ancillary and post-trial care	30	Provisions, if any, for ancillary and post-trial care, and for compensation to those who suffer harm from trial participation	N/A
Dissemination policy	31a	Plans for investigators and sponsor to communicate trial results to participants, healthcare professionals, the public, and other relevant groups (eg, via publication, reporting in results databases, or other data sharing arrangements), including any publication restrictions	22
	31b	Authorship eligibility guidelines and any intended use of professional writers	23
	31c	Plans, if any, for granting public access to the full protocol, participant-level dataset, and statistical code	23
**Appendices**			
Informed consent materials	32	Model consent form and other related documentation given to participants and authorised surrogates	Provided in main protocol not journal

For peer review only - http://bmjopen.bmj.com/site/about/guidelines.xhtml

Biological specimens	33	Plans for collection, laboratory evaluation, and storage of biological specimens for genetic or molecular analysis in the current trial and for future use in ancillary studies, if applicable	N/A

*It is strongly recommended that this checklist be read in conjunction with the SPIRIT 2013 Explanation & Elaboration for important clarification on the items. Amendments to the protocol should be tracked and dated. The SPIRIT checklist is copyrighted by the SPIRIT Group under the Creative Commons "Attribution-NonCommercial-NoDerivs 3.0 Unported" license.

For peer review only

For peer review only - http://bmjopen.bmj.com/site/about/guidelines.xhtml

BMJ Open

Bridging the Age Gap in Breast Cancer: Evaluation of decision support interventions for older women with operable breast cancer: protocol for a cluster randomised controlled trial.

Journal:	*BMJ Open*
Manuscript ID	bmjopen-2016-015133.R2
Article Type:	Protocol
Date Submitted by the Author:	18-May-2017

Complete List of Authors:	Collins, Karen; Sheffield Hallam University, Centre for Health and Social Care Research<br>Reed, Malcolm; Brighton and Sussex Medical School,<br>Lifford, Kate; Cochrane Institute of Primary Care & Public Health,<br>Burton, Maria; Sheffield Hallam University, Centre for Health and Social Care Research<br>Edwards, Adrian; Cardiff University, Division of Population Medicine<br>Ring, Alistair; Royal Marsden NHS Foundation Trust<br>Brain, Kate; Cardiff University, Division of Population Medicine<br>Harder, Helena; University of Sussex, SHORE-C Brighton and Sussex Medical School<br>Robinson, Thomas; University of Leicester, Department of Cardiovascular Sciences<br>Cheung, Kwok; University of Nottingham, School of Medicine<br>Morgan, Jenna; University of Sheffield Medical School, Department of Oncology and Metabolism<br>Audisio, Riccardo; University of Liverpool,<br>Ward, Susan; The University of Sheffield, School of Health and Related Research<br>Paul, Richards; The University of Sheffield, School of Health and Related Research<br>Martin, Charleen; University of Sheffield Medical School, Department of Oncology and Metabolism<br>Chater, Tim; The University of Sheffield, School of Health and Related Research<br>Pemberton, Kirsty; The University of Sheffield, School of Health and Related Research<br>Nettleship, Anthony; The University of Sheffield, School of Health and Related Research<br>Murray, Christopher; The University of Sheffield, School of Health and Related Research<br>Walters, Stephen; University of Sheffield, ScHARR<br>Bortolami, Oscar; The University of Sheffield, School of Health and Related Research<br>Armitage, Fiona; University of Sheffield Medical School, Department of Oncology and Metabolism<br>Robert, Leonard; Imperial College Faculty of Medicine<br>Gath, Jacqui; Independent Cancer Patients' Voice,<br>Revell, Deirdre; The Unviersity of Sheffield, North Trent Cancer Network

	Consumer Research Panel<br>Green, Tracy; The Unviersity of Sheffield, North Trent Cancer Network Consumer Research Panel<br>Wyld, Lynda; University of Sheffield Medical School, Department of Oncology and Metabolism

**Primary Subject Heading**:	Oncology
Secondary Subject Heading:	Health services research, Geriatric medicine
Keywords:	CHEMOTHERAPY, Cancer genetics < GENETICS, GERIATRIC MEDICINE, Risk management < HEALTH SERVICES ADMINISTRATION & MANAGEMENT

SCHOLARONE™ Manuscripts

For peer review only

TITLE: Bridging the Age Gap in Breast Cancer: Evaluation of decision support interventions for older women with operable breast cancer: protocol for a cluster randomised controlled trial.

Corresponding author:
Lynda Wyld,
Department of Oncology & Metabolism
The Medical School
Beech Hill Road
Sheffield
S10 2RX
UK
Email: l.wyld@sheffield.ac.uk
Tel: +44 (0) 114 2259066

Karen Collins, Centre for Health and Social Care Research, Sheffield Hallam University, Collegiate Crescent, Sheffield, UK
Malcolm Reed, Brighton and Sussex Medical School, University of Sussex, Falmer, Brighton, UK.
Kate Lifford, Division of Population Medicine, School of Medicine, Cardiff University, Neuadd Meirionnydd, Heath Park, Cardiff CF14 4YS, UK
Maria Burton, Centre for Health and Social Care Research, Sheffield Hallam University, Collegiate Crescent, Sheffield, UK.
Adrian Edwards, Division of Population Medicine, School of Medicine, Cardiff University, Neuadd Meirionnydd, Heath Park, Cardiff CF14 4YS, UK
Alistair Ring, Royal Marsden Hospital, London.
Katherine Brain, Division of Population Medicine, School of Medicine, Cardiff University, Neuadd Meirionnydd, Heath Park, Cardiff CF14 4YS, UK
Helena Harder, SHORE-C, University of Sussex, Falmer, Brighton, UK.
Thompson Robinson, Department of Cardiovascular Sciences, Leicester Royal Infirmary, Infirmary Square, Leicester, LE2 7LX, UK.
Kwok Leung Cheung, School of Medicine, University of Nottingham, Royal Derby Hospital Centre, Uttoxeter Road, Derby DE22 3DT, UK.
Jenna Morgan, Department of Oncology and Metabolism, University of Sheffield.

For peer review only

Riccardo Audisio, Department of Surgery, University of Liverpool, St Helens Teaching Hospital, Marshalls Cross Road, St Helens, WA9 3DA, UK.

Susan Ward, Department of Health Economics and Decision Science, School for Health and Related Research, University of Sheffield, Sheffield, UK.

Paul Richards, Department of Health Economics and Decision Science, School for Health and Related Research, University of Sheffield, Sheffield, UK.

Charlene Martin, Department of Oncology and Metabolism, University of Sheffield.

Tim Chater, Clinical Trials Research Unit, School for Health and Related Research, University of Sheffield, Sheffield, UK.

Kirsty Pemberton, Clinical Trials Research Unit, School for Health and Related Research, University of Sheffield, Sheffield, UK.

Anthony Nettleship, Epigenesys, School for Health and Related Research, University of Sheffield, Sheffield, UK.

Christopher Murray,Epigenesys, School for Health and Related Research, University of Sheffield, Sheffield, UK.

Stephen Walters, Clinical Trials Research Unit, School for Health and Related Research, University of Sheffield, Sheffield, UK.

Oscar Bortolami, Clinical Trials Research Unit, School for Health and Related Research, University of Sheffield, Sheffield, UK.

Fiona Armitage, University of Sheffield, UK

Robert Leonard, Imperial College Healthcare NHS Trust, St Mary's Hospital London, UK

Jacqui Gath, Deirdre Revell, Tracy Green, Yorkshire and Humberside (formerly North Trent Cancer Network)Consumer Research Panel.

Lynda Wyld, Department of Oncology & Metabolism, University of Sheffield, Sheffield, UK.

Keywords: Breast cancer, decision aid, elderly, primary endocrine therapy, surgery, chemotherapy

Word count excluding title page, abstract, references, figures and tables: 4085/4000

For peer review only

ABSTRACT

Introduction

Whilst breast cancer outcomes are improving steadily in younger women due to advances in screening and improved therapies, there has been little change in outcomes among the older age group. It is inevitable that co-morbidities/frailty rates are higher, which may increase the risks of some breast cancer treatments such as surgery and chemotherapy, many older women are healthy and may benefit from their use. Adjusting treatment regimens appropriately for age/co-morbidity/frailty is variable and largely non-evidence based, specifically with regard to rates of surgery for operable oestrogen receptor (ER) positive disease and rates of chemotherapy for high-risk disease.

Methods and analysis

This multi-centre, parallel group, pragmatic cluster randomised controlled trial (2015-18) reported here, is nested within a larger ongoing "Age Gap Cohort Study" (2012-18; RP-PG-1209-10071), aims to evaluate the effectiveness of a complex intervention of decision support interventions (DESIs) to assist in the treatment decision-making for early breast cancer in older women. The interventions include two patient decision aids (PtDAs) (primary endocrine therapy versus surgery/AET and chemotherapy versus no chemotherapy) and a clinical treatment outcomes algorithm for clinicians.

The primary outcome will be quality of life measured by EORTC QLQ C30. Randomisation is at breast unit level (53 UK sites), stratified by high/ low primary endocrine therapy and chemotherapy rates. Women (n=1500) over 70 years with primary operable breast cancer will be recruited and followed up 6 weeks to 2 years post diagnosis with longer term cancer outcomes (overall survival, disease free survival) derived from cancer registry returns.

For peer review only

Control arm: no change to usual practice. Intervention arm: usual practice plus DESIs adopted as standard care by clinicians.

ETHICS AND DISSEMINATION

National and local ethics committee approval were obtained for all UK participating sites. Results from the trial will be submitted for publication in international peer reviewed scientific journals.

IRAS reference: 115550

Trial registration detail/number:

European Union Drug Regulating Authorities Clinical Trials (EudraCT) number 2015-004220-61

Sponsor's Protocol Code Number Sheffield Teaching Hospitals STH17086

ISRCTN 32447*

*The wider Age Gap study commenced as a cohort study in 2012/13, collecting prospective observational data on older women. At the time there was no requirement for registration on the ISRCTN database as the trial was approved prior to 2013 and was only a cohort study therefore the study team made public notification via the Cancer help database and more recently registered it on the EURDRACT database last year. The trial protocol was changed late 2015/2016 to convert the study to a cluster RCT and at that point registered the revised protocol with the ISRCTN.

For peer review only

Strengths and limitations of this study

- The two evidence based decision support interventions (DESIs) for women over 70 years diagnosed with breast cancer who are offered a choice of primary endocrine therapy (PET) or surgery (plus adjuvant endocrine therapy, hereafter termed surgery/AET) or chemotherapy versus no chemotherapy is, to the best of our knowledge, the first of its kind worldwide.
- The web based clinical outcomes management algorithm is the first of its kind and allows patient age, co-morbidities, frailty and cancer characteristics to be considered in predicting breast cancer survival and cancer outcomes
- A limitations of the trial will potentially be selection bias from recruitment and poor uptake/utilisation of the DESIs at intervention sites
- A second limitation may be an inability to demonstrate a benefit in terms of cancer survival rates without at least 5-10 years follow up or an overall survival advantage due to the competing causes of death in this age group.

For peer review only

INTRODUCTION

Background and rationale

Breast cancer is the most common cancer in women in the UK, with over 53,000 new cases being diagnosed in the UK each year [1].Of these, 16,000 women will be over the age of 70, a figure which is rising steadily as the UK population ages[2]. Whilst breast cancer outcomes are improving steadily in younger women due to advances in screening and improved therapies, there has been little change in outcomes in this older age group of women. The UK lags significantly behind other European countries in its outcomes for these women. There is a wide variation in practice in the management of breast cancer in older women[3]. The gold standard of care for early breast cancer is surgical removal of the primary cancer (mastectomy or conservation surgery), and diagnostic or therapeutic axillary nodal surgery followed by stage and immunophenotype appropriate adjuvant therapies (chemotherapy, trastuzumab, anti-oestrogens and radiotherapy) to reduce the risks of disease recurrence. There is consistent evidence that older women are less likely to receive surgery, chemotherapy, radiotherapy and trastuzumab, based on the premise that there is less evidence of efficacy and a greater risk of treatment morbidity[4]. In the case of surgery, up to 40% of older women do not undergo surgery for their breast cancer, andtheir treatment is mainly with anti-oestrogen tablets alone, known as primary endocrine therapy(PET)[5].Whilst it is inevitable that in older women, co-morbidities and frailty rates are higher, and which will increase the risks of some breast cancer treatments, such as surgery and chemotherapy, many older women are healthy and will benefitin terms of breast cancer outcomes, from their use. Selection of appropriate age, co-morbidity and frailty adjusted treatment regimens is highly variable, largely non-evidence based, and often fails to adequately consider the needs or wishes of patients. Two key areas of local practice variation are rates of surgery for operableoestrogen receptor (ER) positive disease and rates of chemotherapy for high risk

For peer review only

disease. PET rates vary fourfold between UK centres [3] and are not accounted for by case mix adjustment. Similarly rates of chemotherapy vary 10-fold[4].

Recent reports have advocated the use of PET only in the very old or frail[6]. Current national guidelines state that patients with operable breast cancer should be treated with surgery, and not PET, “irrespective of age” unless this is precluded by co-morbidities [7]; whilst the International Society of Geriatric Oncology (SIOG) and European Society of Breast Cancer Specialists (EUSOMA) recommend that PET should only be offered to patients with a “short estimated life expectancy (less than 2 to 3 years), who are considered unfit for surgery… or who refuse surgery”[8]. However, as a large number of older women are treated with PET in UK and other countries, it is not clear whether this guidance is being followed consistently. PET is associated with high rates of patient satisfaction and low treatment morbidity but in the medium and long term some women may need a change of therapy once anti-oestrogen resistance develops [9]. Randomised trials and a recent Cochrane review have shown that surgery (plus adjuvant anti-oestrogens herein after termed surgery/AET) and PET have equivalent overall survival rates [10-11], However, for fitter women with a longer predicted life expectancy, there is evidence that breast cancer specific survival rates are inferior with PET [12]. For very frail women where surgery would be unsafe or poorly tolerated, PET is the clear choice in women with oestrogen sensitive disease [12].

For women at intermediate or higher risk of surgery complications there is a complex series of trade-offs to be made for each patient. The decision must balance the risks of surgical morbidity (pain, risks associated with hospitalisation, surgical complications) but with a greater certainty of local disease control, against the minimal morbidity with PET but a risk of later local disease progression and the need for a change of treatment to either surgery or alternate anti-oestrogen therapy[13-15].

For peer review only

Chemotherapy utilisation is also very low in women over 70 (14%)[4] and almost non-existent in women over 80, even in those where high phenotypic risk is present (high grade, node positive, ER negative, her-2 positive)[4]. Rates of chemotherapy can vary widely between UK breast units, between 6 and 60% in high risk women[16]. This reflects the fact that most of the randomised trials have upper age cut offs at age 70 or recruit very poorly in this age group, meaning there is little evidence of whether it is effective or not. In addition, there is evidence of an increased risk of significant complications such as neutropenic sepsis in older women [17]. This clearly suggests that guidelines for best practice are required. The primary tool used by oncologists to determine the likely benefit of chemotherapy on a patient level basis is Adjuvant!Online[18],although this has been shown to be inaccurate in older women[19].The more recently developed PREDICT tool [20] performs better in this age group but has limited functionality for taking co-morbidity and frailty into account.

This cluster randomised trial will evaluate the implementation of two (“complex”) decision support interventions (DESIs) designed to be used by both clinicians and patients to assist in the decision making about treatment for early breast cancer in older women.

The Bridging the Age Gap Study

The Bridging the Age Gap study[21] is a NIHR funded programme of research (2012-18RP-PG-1209-10071) examining breast cancer management in older women with the ultimate aim of improving outcomes by providing high quality evidence to support treatment decision making in this age group.

The study protocol reported here focuses exclusively on the cluster randomised trial part of the wider Bridging the Age Gap Study [21]. The study group has developed two patient facing decision support interventions (DESIs) based on a systematic evidence summary,expert reference group consultation, patient interviews [22-24] and questionnaires

For peer review only - http://bmjopen.bmj.com/site/about/guidelines.xhtml

about informational needs and preferences and extensive user- and field-testing with both healthy older women and older women who had faced the decision relating to the choice of surgery/AET or PET in frailer women with ER positive breast cancer, and the decision regarding use of adjuvant chemotherapy in fitter women with high risk cancers. Each DESI includes a clinician facing clinical management algorithm and two patient facing decision aids (PtDAs). The clinician facing management algorithms derive from detailed cancer registry outcome data linked to treatment related morbidity and patient and cancer characteristics from the UK cancer registry (2002-2010) for two UK regions (Northern and Yorkshire and East Midlands) which are representative of the UK population as a whole in terms of demography, population structure and deprivation. This is a large diverse area, representing 23% of the UK population [25]. These online algorithms allow patient age, co-morbidities, frailty and cancer characteristics to be considered by a clinician in predicting survival and cancer outcomes and to help inform breast cancer management decisions for older women [25]. The PtDAs are in the form of a booklet and a (brief) option grid for the clinical decision in question [26,27].

The trial will evaluate these tools in a cluster randomised trial across 53 UK breast units according to the study schematic (Figure 1).

The aims of this trial is to evaluate if, how and to what extent, the use of the DESIs embedded as ‘standard of care’ within intervention-arm sites, improves QoL, decision quality (integrating knowledge, attitudes and decision made), coping, illness representations and reduces decision regret, thus indicating improved informed decision making of older women about treatment options for their breast cancer.

To our knowledge this is the first randomised controlled trial to have been undertaken to explore this issue.

For peer review only - http://bmjopen.bmj.com/site/about/guidelines.xhtml

Objectives

The objectives are to:

1. To assess the effectiveness of the implementation of DESIs [26][27] in clinical practice in terms of improving patient QoL, decision quality (integrating knowledge, attitudes and decision made), coping, illness representations and reducing decision regret, thus indicating improved informed decision making.
2. To determine if, how, or to what extent, the clinical outcomes management algorithm impacts on clinical decision making among clinicians (change in PET/surgery rates and chemotherapy rates).
3. To determine whether the DESIs are effective in improving short, medium and long term cancer outcomes in this age group of women, (treatment morbidity and overall and disease specific survival).
4. To assess the utility and uptake of the DESIs from the perspective of both clinicians and patients by undertaking a formal process evaluation.

Hypotheses

1. Use of the DESIs will improve the quality of life in older women with operable breast cancer and ultimately improve cancer outcomes.

For peer review only

2. Older women faced with a choice of treatment decisions for their breast cancer will report an improved decision quality and shared decision-making experience and less decision regret using DESIs compared to older women who receive usual clinical decision making support.
3. Use of evidence based DESIs will improve short and longer term outcomes by improving treatment personalisation to a woman's health, fitness and cancer characteristics and by improving the quality of decision making, reduce the heterogeneity of practice across the UK.
4. Women in the intervention sites will express more positive illness representations (e.g. increased personal control, positive emotional consequences, less overall threat) and increased use of engagement coping strategies compared to women from the control sites.

METHOD

Study design and setting

This protocol follows the CONSORT statement guidelines for cluster trials [28].

This study is a multi-centre, parallel group, pragmatic cluster randomised controlled trial (2015-18) [29]. It is nested within a larger ongoing Bridging the Age Gap Cohort Study (2012-18) [21] (Figure 1) which is currently recruiting from 53 breast units within in the UK (observational cohort study of current UK management of older women with early breast cancer).

The RCT study

For peer review only

The intervention comprises implementation of a package of two DESIs for the PET versus surgery/AET, or chemotherapy versus no chemotherapy decisions. Each DESI includes an online algorithm for treatment outcomes, and two patient decision aids (PtDAs)– a booklet and a brief option grid [26-27]. Each DESI is a complex intervention, including training for the clinician (breast surgeon, medical oncologist, breast care nurses) on the use of the algorithm (surgeons and medical oncologists only) or PtDAs, and the clinician and patient decide which, if any, of these elements they wish to use to assist the decision making process. The intention being for the intervention to be used as part of everyday clinical practice/pathway within the intervention sites.

Each online algorithm includes functionality to adjust outcome prediction according to patient age, co-morbidity, frailty, tumour stage and ER status and which gives outputs of 2 and 5 year overall and breast cancer specific survival. The algorithms were developed in the earlier phase of the Age Gap Study [25] and were designed to guide clinicians and their patients in the treatment of:

(1) frailer older women with ER positive breast cancer to optimise treatment with either PET or surgery/AET,

or

(2) fitter older women who have already had primary surgery and been found to have high risk cancer characteristics (e.g. ER negative, Her 2 positive or node positive breast cancer) to optimise treatment with either adjuvant chemotherapy or no adjuvant chemotherapy (note the term chemotherapy includes chemotherapy +/- trastuzumab if appropriate).

The algorithm is based on a computer model of predicted outcomes and variance caused by patient and disease parameters. Unlike existing web based algorithms for cancer treatment

For peer review only

(Adjuvant! OnLine [19] or PREDICT[20]) which do not have the facility to specify frailty or comorbidity in detail (or at all), the Age Gap algorithm permits these factors to be taken into account. The Age Gap tool has been optimised for accuracy in this age group and has been based on analysis of data from over 20 000 UK women over the age of 70 derived from cancer registry data.The algorithm has built in educational materials (including several on line presentations, data sources, FAQs and an animated educational video). The online algorithm is designed to be used by clinicians to guide treatment decision making and its outputs can be printed off in a patient facing format that could be used in personalised patient counselling. The report provides specific survival estimates for each treatment option for an individual woman based on her personal and cancer characteristics. This works in much the same way as the print outs from Adjuvant!Online[19] or PREDICT[20] but in this case developed for the PET versus surgery/AET decision and with more detailed data entry relating to the woman's age and fitness level.

Two PtDAs (PET versus surgery/AET[26] and chemotherapy versus no chemotherapy [27]) have been developed during the earlier phase of the study [22-24]. The PtDAs comprise of an option grid [30] and a booklet for each decision. The option grid is a one page evidence-based summary of the treatment options alongside patients' frequently asked questions, helping patients to differentiate the key features, risks and benefits of treatment options in relation to their personal values and preferences. The option grid has been designed to be sufficiently brief for use in clinical encounters and accessible enough to support a better dialogue between patients and their clinical team [30]. The booklet provides information about both options including diagrams, side effects and potential risks and benefits. It also includes a section to guide deliberation and encourage the patient to clarify their preferences based around identifying "what is most important to them" [16].

For peer review only

Eligibility criteria

Inclusion criteria

(1) Female

(2) Aged over 70 years of age at the time of diagnosis of cancer

(3) Primary operable (TNM categories V7: T1, T2, T3, N0, N1, M0), ER positive invasive breast cancer (core biopsy or diagnostic incision biopsy)

(4) Ability to give informed consent and to read English

Exclusion criteria

(1) Disease unsuitable for surgery e.g. inoperable, locally recurrent or metastatic disease.

(2) Previous invasive breast cancer within the last 5 years.

(3) Non-English speakers

Data Collection and Outcomes.

Primary outcome measure

The primary outcome measure for the RCT is global health status/QoL score (questions 29+30 only of The European Organization for Research and Treatment of Cancer QLQ-C30 Reference Manual) (EORTC QLQ-C30) [31]. This primary end point was stipulated by the funder of the study with the justification being that the EORTC QLQ-C30 is internationally recognised and well validated QoL measure (as opposed to our original primary endpoint of decision quality). This was measured at 6 weeks and 6 months post diagnosis/consent.

For peer review only

An independent data monitoring committee (DMC) comprising of 3 experienced academic clinicians oversees the study and monitors trial conduct and safety and potential harm and has access to all study data. The role being to provide recommendations for trial changes (or closure). Data collection is being undertaken by trained clinical staff within each of the participating sites. The study data manager and study monitor also undertake regular site visits to outline the study protocol, ensure protocol adherence and monitor data collection and completeness. Data collection for the study includes detailed information about the patient and their cancer at the time of diagnosis: age, comorbidity (Charlson co-morbidity index [32], frailty- The Barthel Index (ADL) [33] and instrumental activities of daily livingscores (IADL) [34]), cognitive status (Mini-mental state examination-MMSE) [35], baseline QoL (EORTC QLQ C30 [31], EORTC breast cancer-specific QoL questionnaire (QLQ-BR23) [36], EORTC QoL questionnaire module for older people with cancer(QLQ-ELD14) [37], EuroQol Group EQ-5D[38]), tumour stage, grade and receptor status. Treatment details are recorded including the type of surgery to the breast and axilla, use of adjuvant therapies (chemotherapy, radiotherapy, trastuzumab and hormonal therapies), including doses and adverse effects recorded using the Common Terminology Criteria for Adverse Events grading system. Follow up is at baseline, 6 weeks, 6, 12, 18 and 24 months after diagnosis/consent. Cancer outcomes, QoL and adverse events are recorded at each visit and in the longer term, women are asked to sign a consent form to permit the trial to collect their Cancer Registry data which will be collected 5 and 10 years following diagnosis and consent to the study. These data will permit us to look at whether using the DESIs alters patterns of treatment decision making between control and intervention sites and whether these impact on long term outcomes. As such this is a uniquely detailed evaluation of such DESIs.

In addition, specific questionnaires relating to patient choice and decision making will be administered. These will apply to all women offered a choice of either PET and surgery/AET

For peer review only

or chemotherapy versus no chemotherapy and are administered in relation to the time of their treatment choice. Secondary outcomes measures here include decision regret (Decision Regret Scale [39], shared decision making (CollaboRATE [40]), patient anxiety (Spielberger short-form State scale of the State-Trait Anxiety Inventory[41], knowledge and preference (knowledge,readiness to decide and preference measure[42-43]), illness perceptions (Brief Illness Perceptions Questionnaire [44]) and Coping(brief COPE)[45]). Original data collected are entered and kept on file within each of the study sites. This data is entered electronically and stored securely onto password protected databases within local databases and the main trial office. All records that contain names or other personal identifiers, such as locator forms and informed consent forms, are stored separately from study records identified by code number. Only the study steering and DMC have access to the full trial dataset Errors, discrepancies or missing data are captured by the computer programme and the study data manager checks and subsequently follows this up with participating sites.

The timescales for each of these are shown in Table 1.

Table 1.Data items relating to patient-based outcomes and cancer characteristics.

For peer review only

Table 1: Questionnaire Schedule

**Standard Age Gap Questionnaires**	**Baseline**	**6 weeks**	**6mths**	**12mths**	**18/24 mths**	**Long-term**
IADL	*					
ADL	*					
MMSE	*					
ECOG perf. status	*					
Subjective Global Assessment	*					
Co-morbidity	*					
EQ5D	*	*	*	*	*	
QoL (EORTC-QLQ C30; QLQ-BR23 and (QLQ-ELD14)	*	*	*	*	*	
Decision quality	*	*				
RECIST if PET	*	*	*	*	*	
Registry data access						*
Tissue Access	*					*
Tumour details	*					
Treatment details	*	*	*	*	*	
Adverse events	*	*	*	*	*	
**New for DESI study** (if offered choice of either PETor surgery/AET, or chemotherapy/no chemotherapy)	Baseline (after consent for PET or surgery (AET or after consultation for chemo/no chemo, as applicable)	6 weeks after relevant treatment choice	6 months after relevant treatment choice			
Spielberger Anxiety		*	*			
Collaborate	*					
Decision Regret		*	*			
Knowledgereadiness to decide and preference measures	*					
Brief IPQ		*	*			
Brief COPE		*	*			
**Process evaluation** (if taking part in process evaluation)						
Process evaluation questionnaire		*				

Sample size calculation

The primary endpoint will be the global health status/QoL scale (questions 29 and 30 of the EORTC-QLQ-C30)[31] at 6 months post baseline. Assuming a SD of 21 points for the global health status/QoL scale and a mean difference of 7 or more points on the global health status/QoL scale between the groups is of clinical/practical importance (a "small" standardised effect size of 0.33). With no allowance for clustering; for the PET versus surgery DESI comparison with 190 eligible women per group we will have a 90% power of detecting this difference or more as statistically significant between the groups at the 5% two-sided level. If we assume an intra-class correlation of 0.03 then allowing for the clustered RCT design we will need to recruit 10 women, eligible for using the decision aids, per cluster (i.e. 50 clusters x 10 women), 500 in total (this assumes a design effect of 1.3). With a 20% loss to follow-up by 6 months we need to recruit 13 women per cluster (50 clusters x 13 women) or 650 in total (325 per group).

Randomisation

Randomisation is at breast unit level, stratified by high and low PET and chemotherapy rates. It was therefore not possible to blind the investigators or the study sites to the allocation of participants. Data for this stratification have been derived from the wider cohort study which has collected data on treatment rates for both PET versus surgery/AET and chemotherapy versus no chemotherapy.

Control arm. Usual standard practice for older women (>70 years) diagnosed with breast cancer with no change to normal treatment decision making practice.

For peer review only

Intervention arm. Usual standard practice for older women (>70 years) diagnosed with breast cancer plus optional clinician and patient access to the package of DESIs which will have been made available to these units to adopt as their standard of care.

In the run in to the trial period (June–Dec 2015), clinical teams (clinicians, research and breast nurses) from the participating sites attended a training event to enhance concordance with the study protocol (control group) and provide additional training on shared decision making and the use of the DESIs (intervention group). This comprised of a 2 hour practical workshop which consisted of presentations, demonstrations and discussion based on the MAGIC programme [46].

Recruitment

Potentially eligible women are identified by clinicians (breast surgeons, medical oncologists and specialist breast nurses) and research nursing staff within multi disciplinary teams of the study sites. Study packs are being given to eligible patients either following their clinical consultation where either PET or surgery/AET options or chemotherapy versus no chemotherapy options are discussed. Monthly study newsletters are sent to all participating sites to provide feedback to staff in order to maintain interest and recruitment to the study. Any modifications to the original study protocol will be discussed with the DMEC and approvals sought from the funder and the ethics committee. Recruitment for the trial has now commenced and 750 women have been recruited over the 53 participating sites.

Data analysis

The statistical analyses will be performed on an intention-to-treat basis comparing the DESI and control groups. All statistical exploratory tests will be two-tailed with p= 0.05. Baseline

demographic (e.g. age), physical measurements, and health-related QoL data will be assessed for comparability between the treatment groups. A marginal Generalised Linear Model (GLM), with coefficients estimated using generalised estimating equations (GEE) with robust standard errors and an exchangeable auto correlation matrix in STATA will be used to analyse the outcomes and allow for the clustered nature of the data. The exchangeable correlation structure corresponds to an equal correlation model, meaning that the correlations of the outcomes with a cluster, i.e. breast centres, are the same. For continuous outcomes, such as mean global health status/QoL score (questions 29+30 of EORTC QLQ-C30 [31]) at 6 months post-diagnosis/consent intervention, knowledge score and preference for treatment score, an identity link with a Normal distribution for the outcome will be used. Estimates for the treatment group coefficient from this regression model will be reported along with their associated 95% confidence interval. In the event of differences between the intervention and control groups with respect to baseline demographic, physical, and health-related QoL measurements, then these covariates will be used in the GLM to adjust the treatment effect for these variables. The adjusted regression coefficient estimate for the treatment group parameter along with its 95% confidence interval (CI) will then be reported.

For the other secondary outcomes, at 6 weeks and 6 months, such as the other dimensions of the EORTC QLQ C30 [31], the EORTC QLQ-BR23 [36] and EORTC QLQ-ELD14 [37] the mean QoL dimension scores will be compared between the intervention and control groups, using similar models.

A series of exploratory sub group analyses using a marginal GLM with coefficients estimated using GEE with robust standard errors and an exchangeable auto correlation matrix, with the primary outcome the mean Global health status/QoL score (questions 29+30 of EORTC QLQ-C30 [31]) at 6-month post-diagnosis/consent randomisation as the response will be carried out. An interaction statistical test between the randomised intervention group and

For peer review only

subgroup to directly examine the strength of evidence for the treatment difference between the treatment groups (Intervention versus Control) varying between subgroups will be undertaken. Age subgroup (75-79, 80-84, 85-89 and 90+ years) and co-morbidity levels (based on the modified Charlson co-morbidity score [32]) will be the only a priori defined sub groups to be considered for interaction test. Sub group analysis will be performed regardless of the statistical significance on the overall intervention effect (intervention versus control).

Missing primary outcome data

A sensitivity analysis using a variety of imputation methods, to impute any missing primary outcome data (6-month EORTC QLQ-C30 [31] global health status/QoL score) will be performed. The imputation methods will include last observation carried forward, regression and multiple imputation. The estimates of the treatment effect and its associated confidence interval, from the various imputation methods, will be graphically displayed alongside the results for the observed data.

Process Evaluation

Running alongside the main study, a detailed mixed methods process evaluation is being undertaken at 16 sites to assess the implementation of the DESIs(fidelity to the trial protocol) to consider the DESIs' usefulness and acceptability and examine the facilitators and barriers to embedding them into everyday clinical practice. A random selection of breast units was made stratified by trial arm and recruitment rate to the cohort study (high/low PET/surgery/chemo rates).

In summary, the Age Gap study [21] aims to improving outcomes of older women diagnosed with breast cancer by providing high quality evidence to support treatment decision making

For peer review only

in this age group. The two evidence based DESIs each include a clinical management algorithm and two patient decision aids (PtDAs) in the form of a booklet and a (brief) option grid for the clinical decision in question. These online algorithms will allow patient age, co-morbidities, frailty and cancer characteristics to be considered by a clinician in predicting survival and cancer outcomes and to help inform breast cancer management decisions for older women.

Acknowledgments

The authors are grateful to all participating sites, and specifically site principal investigators (PI) who have or who are currently contributing to the RCT

Competing interests: None declared

Funding: This work is supported by National Institute of Health Research Programme Grant [grant number RP-PG-1209-10071]

Contributors LW, KC and MR were overall project leads. Decision aid development and testing KL, MB, KC, LW, MR, AE, KB, JG, DR, FA, HH. Trial management: LW, CM, TC, KP. On line tool development: LW, SW, PR, AN, CM, MB, JM. Statistical advice: SW, OB. Trial management group: LW, MR, KC, TR, KLC, AR, etc. Chemotherapy advisors: AR, RL, HH. All authors have contributed to reading and approved the final manuscript.

Provenance and peer review: Not commissioned; externally peer reviewed

Open access: This is an Open Access article distributed in accordance with the Creative Commons Attribution Non Commercial (CC BY-NC 4.0) license, which permits others to distribute, remix, adapt, build upon this work non-commercially, and license their derivative works on different terms, provided the original work is properly cited and the use is non-commercial. See: http://creativecommons.org/licenses/by-nc/4.0/

For peer review only

REFERENCES

1. Cancer Research UK breast cancer incidence [accessed 1/11/2016] Available from http://www.cancerresearchuk.org/health-professional/cancer-statistics/statistics-by-cancer-type/breast-cancer
2. Office for National Statistics. New cases of cancer diagnosed in England, 2014: selected sites by age group and sex. Office for National Statistics 2010[accessed 1/11/2016]Availablefromhttps://www.ons.gov.uk/peoplepopulationandcommunity/birthsdeathsandmarriages/deaths/bulletins/deathsregistrationsummarytables/previousReleases
3. Morgan J, Richards P, Ward S et al (2015) Case mix analysis and variation in rates of non-surgical treatment of older women with operable breast cancer. *British Journal of Surgery.* 102 (9), 1056-1063.
4. Ring A, Harder H, Langridge C et al. Adjuvant chemotherapy in elderly women with breast cancer (AChEW): an observational study identifying MDT perceptions and barriers to decision making. Ann Oncol. 2013;24(5):1211-9.
5. BCCOM. Breast Cancer Clinical Outcome Measures (BCCOM) Project. Birmingham, UK; 2007
6. Chakrabarti J, Kenny FS, Syed BM, et al. A randomised trial of mastectomy only versus tamoxifen for treating elderly patients with operable primary breast cancer-final results at 20-year follow-up. Crit Rev OncolHematol 2011;78:260-264.
7. NICE. Early and locally advanced breast cancer: full guideline.Cardiff: National Collaborating Centre for Cancer; 2009.
8. Biganzoli L, Wildiers H, Oakman C. et al. Management of elderly patients with breast cancer: updated recommendations of the International Society of Geriatric Oncology

eview only

(SIOG) and European Society of Breast Cancer Specialists (EUSOMA). Lancet Oncol 2012;13:e148-e160.

9. Morgan JL, Reed MW, Wyld L. Primary endocrine therapy as a treatment for older women with operable breast cancer - a comparison of randomised controlled trial and cohort study findings. Eur J Surg Oncol. 2014;40(6):676-84.

10. Glas de, NA, Jonker JM, Bastiaannet E, et al. Impact of omission of surgery on survival of older patients with breast cancer. Br J Surg. 2014;101(11):1397-404.

11. Hind D, Wyld L, Reed MW. Surgery, with or without tamoxifen, vs tamoxifen alone for older women with operable breast cancer: cochrane review. Br J Cancer. 2007;96(7):1025-9.

12. Morgan J, Wyld L, Collins K, et al. Surgery versus primary endocrine therapy for operable primary breast cancer in elderlywomen (70 years plus). Cochrane Db Syst Rev 2014;5:CD004272.

13. Audisio RA, Wyld L. No standard is set for older women with breast cancer. Eur J SurgOncol 2015 May; 41 (5): 607

14. Morgan JL, Reed MW, Wyld L. Primary endocrine therapy as a treatment for older women with operable breast cancer - a comparison of randomised controlled trial and cohort study findings. Eur J SurgOncol. 2014;40(6):676-84.

15. Hille U, Soergel P, Länger F, et al. Aromatase inhibitorsas solely treatment in postmenopausal breast cancer patients. Breast J 2012;18(2):145–50.

16. Elwyn G, Frosch D, Thomson R et al. Shared decision making: a model for clinical practice. J Gen Intern Med 2012 Oct; 27 (10): 1361-7.

17. NICE Clinical guideline. Neutropenic sepsis: prevention and management in people with cancer. CG151]Published date: September 2012

For peer review only

18. Ravdin PM, Siminoff LA, Davis GJ, et al. Computer program to assist in making decisions about adjuvant therapy for women with early breast cancer. J Clin Oncol. 2001;19(4):980-91.

19. deGlas NA, van de Water W, Engelhardt EG, et al. Validity of Adjuvant! Online program in older patients with breast cancer: a population-based study. Lancet Oncol. 2014;15(7):722-9.

20. Wishart GC, Azzato EM, Greenberg DC, et al. PREDICT: a new UK prognostic model that predicts survival following surgery for invasive breast cancer.*Breast Cancer Res. 2010*; 12(1): R1. Published online 2010 January 6. doi: 10.1186/bcr2464. PMCID: PMC2880419

21. Wyld L, Reed MWR, Collins K et al .Bridging the Age Gap in Breast Cancer: Improving outcomes for older women NIHR Programme grant 2012-17RP-PG-1209-10071[Accessed 18/11/ 2016] Available athttp://www.nihr.ac.uk/funding/funded-research/funded-research.htm?postid=2237.

22. Lifford KJ, Witt J, Burton M, et al. Understanding older women's decision making and coping in the context of breast cancer treatment. *BMC Medical Informatics & Decision Making* 2015. 15:45.

23. Burton M,Collins K, Caldon LJM et al.Information Needs of Older Women Faced With a Choice of Primary Endocrine Therapy or Surgery for Early Stage Breast Cancer: A Literature Review; Current Breast Cancer Reports.2014; 6:235–244.

24. Burton M, Collins K, Lifford K et al.The information and decision support needs of older women (>75 yrs) facing treatment choices for breast cancer: a qualitative study (2014). Psycho-Oncology. Published online 22 December 2014.

For peer review only

25. Richards P, Ward S, Morgan J, et al. The use of surgery in the treatment of ER+ early stage breast cancer in England: variation by time, age and patient characteristics. European Journal of Surgical Oncology. 2016; 42(4) 489-96.

26. Deciding about your breast cancer treatment: surgery and hormone-blocking pills or hormone-blocking pills only. Cardiff University, The University of Sheffield, Sheffield Hallam University developed as part of the Bridging the Age Gap Study (RP-PG-1209-10071) August 2015.

27. Deciding about your breast cancer treatment: chemotherapy or no chemotherapy. The University of Sheffield ,Brighton and Sussex Medical School, Sheffield Hallam University and Cardiff Universityas part of the Bridging the Age Gap Study (RP-PG-1209-10071) January 2016.

28. Schulz KF, Altman DG, Moher D, Group C. CONSORT 2010 statement: updated guidelines for reporting parallel group randomized trials. Ann Intern Med. 2010;152(11):726-32.

29. Wyld L, Reed MWR, Collins K on behalf of the Age Gap study. A multi-centre, parallel group, pragmatic cluster randomised controlled trial NIHR Programme grant RP-PG-1209-1007.EUDRACT No:2015-004220-61

30. Marrin k, Brain K, Durand MA, Edwards A et al. . Fast and frugal tools for shared decsion making: How to develop option grids.European Journal for Person Centered Health Care. 2013;1(1):240-5.

31. Aaronson NK, Ahmedzai S, Bergman B, et al. The European Organization for Research and Treatment of Cancer QLQ-C30: a quality-of-life instrument for use in international clinical trials in oncology. J Natl Cancer Inst. 1993;85(5):365-76.

For peer review only

32. Charlson ME, Pompei P, Ales KL, MacKenzie CR. A new method of classifying prognostic comorbidity in longitudinal studies: development and validation. J Chronic Dis. 1987;40(5):373-83.

33. Mahoney FI, Barthel DW. Functional Evaluation: The Barthel Index. Md State Med J. 1965;14:61-5.

34. Lawton MP, Brody EM. Assessment of older people: self-maintaining and instrumental activities of daily living. Gerontologist. 1969;9(3):179-86.

35. Folstein MF, Folstein SE, McHugh PR. "Mini-mental state". A practical method for grading the cognitive state of patients for the clinician. J Psychiatr Res. 1975;12(3):189-98.

36. Sprangers MA, Groenvold M, Arraras JI et al. The European Organization for Research and Treatment of Cancer breast cancer-specific quality-of-life questionnaire module: first results from a three-country field study. J Clin Oncol. 1996;14(10):2756-68.

37. Johnson C, Fitzsimmons D, Gilbert J et al. Development of the European Organisation for Research and Treatment of Cancer quality of life questionnaire module for older people with cancer: The EORTC QLQ-ELD14. Eur J Cancer. 2010;46(12):2242-52.

38. EuroQol Group EuroQol—A new facility for the measurement of health-related quality of life

39. Health Policy, 16 (1990), pp. 199–208.Brehaut JC, O'Connor AM, Wood TJ, Hack TF, Siminoff L, Gordon E, et al. Validation of a decision regret scale. Med Decis Making. 2003;23(4):281-92.

For peer review only

40. Barr PJ, Thompson R, Walsh T, et al. The psychometric properties of CollaboRATE: a fast and frugal patient-reported measure of the shared decision-making process. J Med Internet Res. 2014;16(1):e2.

41. Marteau TM, Bekker H. The development of a six-item short-form of the state scale of the Spielberger State-Trait Anxiety Inventory (STAI). Br J Clin Psychol. 1992;31 (Pt 3):301-6.

42. Sepucha K, Ozanne E, Silvia K, et al. An approach to measuring the quality of breast cancer decisions. Patient Educ Couns. 2007;65(2):261-9.

43. Sepucha KR, Belkora JK, Chang Y, et al. Measuring decision quality: psychometric evaluation of a new instrument for breast cancer surgery. BMC Med Inform Decis Mak. 2012;12:51.

44. Broadbent E, Petrie KJ, Main J, et al. The brief illness perception questionnaire. J Psychosom Res. 2006;60(6):631-7.

45. Carver CS. You want to measure coping but your protocol's too long: Consider the Brief COPE. International Journal of Behavioral Medicine.1997; 4, 92-100. .

46. Lloyd A & Joseph-Williams N. Case Study: Different outcomes from different approaches – Experience from the Cardiff MAGIC Programme, UK. In Elwyn G, Edwards A, Thompson R (eds.) Shared Decision Making in Health Care. 3rd edition. 2016. Oxford: Oxford University Press.

For peer review only

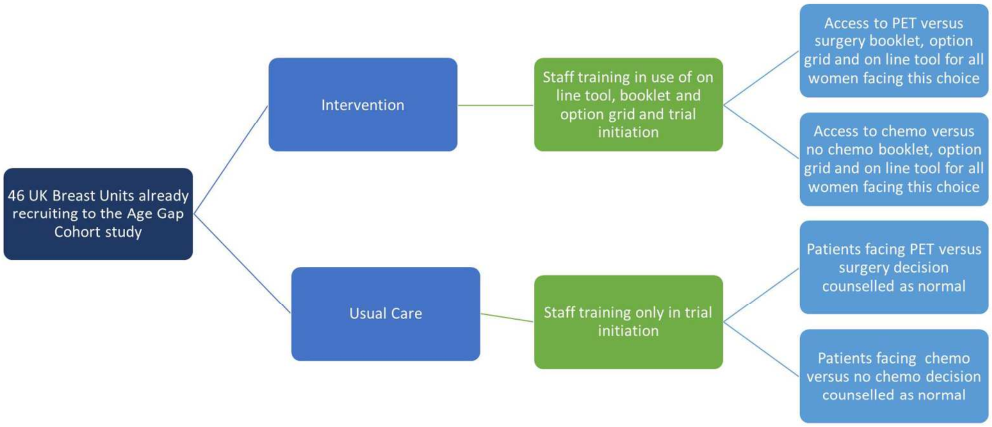

Figure 1: Overview of the Cluster randomised Controlled Trial

335x182mm (300 x 300 DPI)

For peer review only - http://bmjopen.bmj.com/site/about/guidelines.xhtml

SPIRIT
STANDARD PROTOCOL ITEMS: RECOMMENDATIONS FOR INTERVENTIONAL TRIALS

SPIRIT 2013 Checklist: Recommended items to address in a clinical trial protocol and related documents*

Section/item	Item No	Description	Addressed on page number
**Administrative information**			
Title	1	Descriptive title identifying the study design, population, interventions, and, if applicable, trial acronym	1
Trial registration	2a	Trial identifier and registry name. If not yet registered, name of intended registry	4
	2b	All items from the World Health Organization Trial Registration Data Set	Within main protocol but N/A for journal
Protocol version	3	Date and version identifier	Within main protocol but N/A for journal
Funding	4	Sources and types of financial, material, and other support	23
Roles and responsibilities	5a	Names, affiliations, and roles of protocol contributors	23
	5b	Name and contact information for the trial sponsor	4
	5c	Role of study sponsor and funders, if any, in study design; collection, management, analysis, and interpretation of data; writing of the report; and the decision to submit the report for publication, including whether they will have ultimate authority over any of these activities	4

For peer review only - http://bmjopen.bmj.com/site/about/guidelines.xhtml

For peer review only

	5d	Composition, roles, and responsibilities of the coordinating centre, steering committee, endpoint adjudication committee, data management team, and other individuals or groups overseeing the trial, if applicable (see Item 21a for data monitoring committee)	Within main protocol but N/A for journal
**Introduction**			
Background and rationale	6a	Description of research question and justification for undertaking the trial, including summary of relevant studies (published and unpublished) examining benefits and harms for each intervention	6-8
	6b	Explanation for choice of comparators	18,19
Objectives	7	Specific objectives or hypotheses	10-11
Trial design	8	Description of trial design including type of trial (eg, parallel group, crossover, factorial, single group), allocation ratio, and framework (eg, superiority, equivalence, noninferiority, exploratory)	11
**Methods: Participants, interventions, and outcomes**			
Study setting	9	Description of study settings (eg, community clinic, academic hospital) and list of countries where data will be collected. Reference to where list of study sites can be obtained	11
Eligibility criteria	10	Inclusion and exclusion criteria for participants. If applicable, eligibility criteria for study centres and individuals who will perform the interventions (eg, surgeons, psychotherapists)	14
Interventions	11a	Interventions for each group with sufficient detail to allow replication, including how and when they will be administered	12-13
	11b	Criteria for discontinuing or modifying allocated interventions for a given trial participant (eg, drug dose change in response to harms, participant request, or improving/worsening disease)	15
	11c	Strategies to improve adherence to intervention protocols, and any procedures for monitoring adherence (eg, drug tablet return, laboratory tests)	19
	11d	Relevant concomitant care and interventions that are permitted or prohibited during the trial	N/A

For peer review only - http://bmjopen.bmj.com/site/about/guidelines.xhtml

Outcomes	12	Primary, secondary, and other outcomes, including the specific measurement variable (eg, systolic blood pressure), analysis metric (eg, change from baseline, final value, time to event), method of aggregation (eg, median, proportion), and time point for each outcome. Explanation of the clinical relevance of chosen efficacy and harm outcomes is strongly recommended	15-17
Participant timeline	13	Time schedule of enrolment, interventions (including any run-ins and washouts), assessments, and visits for participants. A schematic diagram is highly recommended (see Figure)	17
Sample size	14	Estimated number of participants needed to achieve study objectives and how it was determined, including clinical and statistical assumptions supporting any sample size calculations	18
Recruitment	15	Strategies for achieving adequate participant enrolment to reach target sample size	19
**Methods: Assignment of interventions (for controlled trials)**			
Allocation:			
Sequence generation	16a	Method of generating the allocation sequence (eg, computer-generated random numbers), and list of any factors for stratification. To reduce predictability of a random sequence, details of any planned restriction (eg, blocking) should be provided in a separate document that is unavailable to those who enrol participants or assign interventions	18
Allocation concealment mechanism	16b	Mechanism of implementing the allocation sequence (eg, central telephone; sequentially numbered, opaque, sealed envelopes), describing any steps to conceal the sequence until interventions are assigned	N/A 18
Implementation	16c	Who will generate the allocation sequence, who will enrol participants, and who will assign participants to interventions	18
Blinding (masking)	17a	Who will be blinded after assignment to interventions (eg, trial participants, care providers, outcome assessors, data analysts), and how	N/A 18
	17b	If blinded, circumstances under which unblinding is permissible, and procedure for revealing a participant’s allocated intervention during the trial	N/A
**Methods: Data collection, management, and analysis**			

For peer review only - http://bmjopen.bmj.com/site/about/guidelines.xhtml

Data collection methods	18a	Plans for assessment and collection of outcome, baseline, and other trial data, including any related processes to promote data quality (eg, duplicate measurements, training of assessors) and a description of study instruments (eg, questionnaires, laboratory tests) along with their reliability and validity, if known. Reference to where data collection forms can be found, if not in the protocol	14-16
	18b	Plans to promote participant retention and complete follow-up, including list of any outcome data to be collected for participants who discontinue or deviate from intervention protocols	17
Data management	19	Plans for data entry, coding, security, and storage, including any related processes to promote data quality (eg, double data entry; range checks for data values). Reference to where details of data management procedures can be found, if not in the protocol	16
Statistical methods	20a	Statistical methods for analysing primary and secondary outcomes. Reference to where other details of the statistical analysis plan can be found, if not in the protocol	19-21
	20b	Methods for any additional analyses (eg, subgroup and adjusted analyses)	21
	20c	Definition of analysis population relating to protocol non-adherence (eg, as randomised analysis), and any statistical methods to handle missing data (eg, multiple imputation)	21
**Methods: Monitoring**			
Data monitoring	21a	Composition of data monitoring committee (DMC); summary of its role and reporting structure; statement of whether it is independent from the sponsor and competing interests; and reference to where further details about its charter can be found, if not in the protocol. Alternatively, an explanation of why a DMC is not needed	15
	21b	Description of any interim analyses and stopping guidelines, including who will have access to these interim results and make the final decision to terminate the trial	17
Harms	22	Plans for collecting, assessing, reporting, and managing solicited and spontaneously reported adverse events and other unintended effects of trial interventions or trial conduct	15
Auditing	23	Frequency and procedures for auditing trial conduct, if any, and whether the process will be independent from investigators and the sponsor	15,17
**Ethics and dissemination**			

For peer review only - http://bmjopen.bmj.com/site/about/guidelines.xhtml

Research ethics approval	24	Plans for seeking research ethics committee/institutional review board (REC/IRB) approval	22
Protocol amendments	25	Plans for communicating important protocol modifications (eg, changes to eligibility criteria, outcomes, analyses) to relevant parties (eg, investigators, REC/IRBs, trial participants, trial registries, journals, regulators)	15
Consent or assent	26a	Who will obtain informed consent or assent from potential trial participants or authorised surrogates, and how (see Item 32)	19
	26b	Additional consent provisions for collection and use of participant data and biological specimens in ancillary studies, if applicable	N/A
Confidentiality	27	How personal information about potential and enrolled participants will be collected, shared, and maintained in order to protect confidentiality before, during, and after the trial	16
Declaration of interests	28	Financial and other competing interests for principal investigators for the overall trial and each study site	23
Access to data	29	Statement of who will have access to the final trial dataset, and disclosure of contractual agreements that limit such access for investigators	15
Ancillary and post-trial care	30	Provisions, if any, for ancillary and post-trial care, and for compensation to those who suffer harm from trial participation	N/A
Dissemination policy	31a	Plans for investigators and sponsor to communicate trial results to participants, healthcare professionals, the public, and other relevant groups (eg, via publication, reporting in results databases, or other data sharing arrangements), including any publication restrictions	22
	31b	Authorship eligibility guidelines and any intended use of professional writers	23
	31c	Plans, if any, for granting public access to the full protocol, participant-level dataset, and statistical code	23
**Appendices**			
Informed consent materials	32	Model consent form and other related documentation given to participants and authorised surrogates	Provided in main protocol not journal

For peer review only - http://bmjopen.bmj.com/site/about/guidelines.xhtml

Biological specimens	33	Plans for collection, laboratory evaluation, and storage of biological specimens for genetic or molecular analysis in the current trial and for future use in ancillary studies, if applicable	N/A

*It is strongly recommended that this checklist be read in conjunction with the SPIRIT 2013 Explanation & Elaboration for important clarification on the items. Amendments to the protocol should be tracked and dated. The SPIRIT checklist is copyrighted by the SPIRIT Group under the Creative Commons "Attribution-NonCommercial-NoDerivs 3.0 Unported" license.

For peer review only
